# Firstline treatment with ruxolitinib versus best available therapy in patients with polycythemia vera: results from the randomized RuxoBEAT trial

**DOI:** 10.1038/s41408-026-01644-2

**Published:** 2026-10-22

**Authors:** Steffen Koschmieder, Susanne Isfort, Lino L. Teichmann, Philippe Schafhausen, Florian H. Heidel, Carl Crodel, Andreas Hochhaus, Martin Griesshammer, Katja Sockel, Philipp J. Jost, Denise Walther, Konstanze Döhner, Joachim R. Göthert, Markus Radsak, Marinela Augustin, Heiko Becker, Aristoteles Giagounidis, Markus Schaich, Martine Klausmann, Nikolas von Bubnoff, Frank Stegelmann, Maike Kortmann, Michelle Zentis, Julia Frank, Anna Hagemeier, Martin Hellmich, Tim H. Brümmendorf

**Affiliations:** 1 Department of Medicine (Hematology, Oncology, Hemostaseology, and SCT), Faculty of Medicine, RWTH Aachen University, Aachen, Germany; 2 Center for Integrated Oncology Aachen Bonn Cologne Düsseldorf (CIO ABCD), Aachen, Germany; 3 Hematology, Hemostasis, Oncology and Stem Cell Transplantation, Hannover Medical School (MHH), Hannover, Germany; 4 Department of Medicine III, University Hospital Bonn, Bonn, Germany; 5 Department of Oncology, Hematology, and Bone Marrow Transplantation with Section of Pneumology, University Medical Center Hamburg-Eppendorf, Hamburg, Germany; 6 Clinic for Internal Medicine 2, Jena University Hospital, Jena, Germany; 7 University Clinic for Haematology, Oncology, Haemostaseology and Palliative Care, Johannes Wesling Medical Centre Minden, University of Bochum, Minden, Germany; 8 Medical Clinic and Policlinic I, University Hospital Dresden, TU Dresden, Germany; 9 Medical Department III, Hematology and Oncology, and Center for Translational Cancer Research, Translatum, Technical University of Munich, Munich, Germany; 10 Division of Clinical Oncology, Internal Medicine, Medical University of Graz, Graz, Austria; 11 Department of Hematology/Oncology and Cell Therapy, Otto-von-Guericke University Magdeburg, Medical Center, Magdeburg, Germany; 12 Department of Internal Medicine III, University Hospital of Ulm, Ulm, Germany; 13 Department of Hematology and Stem Cell Transplantation, West German Cancer Center, University Hospital Essen, Essen, Germany; 14 Department of Hematology and Medical Oncology, UCT Mainz, DKTK, University Medical Center, Johannes Gutenberg University Mainz, Mainz, Germany; 15 Klinikum Nürnberg Nord, Klinik für Innere Medizin 5, Schwerpunkt Onkologie/Hämatologie, Paracelsus Medizinische Privatuniversität, Campus Nürnberg, Nürnberg, Germany; 16 Department of Hematology, Oncology and Stem Cell Transplantation, Medical Center, Faculty of Medicine, German Cancer Consortium (DKTK), partner site Freiburg, University of Freiburg, Freiburg, Germany; 17 Dept. Oncology, Hematology and Palliative Care, Marienhospital Düsseldorf, Düsseldorf, Germany; 18 Department of Hematology, Oncology and Palliative Medicine, Rems-Murr-Klinikum, Winnenden, Germany; 19 Gemeinschaftspraxis, Aschaffenburg, Germany; 20 Department of Hematology and Oncology, University Medical Center Schleswig-Holstein and University Cancer Center Schleswig-Holstein (UCCSH), University of Lübeck, Lübeck, Germany; 21 Center for Translational & Clinical Research (CTC-A), Faculty of Medicine, RWTH Aachen University, Aachen, Germany; 22 Institute of Medical Statistics and Computational Biology, Faculty of Medicine and University Hospital Cologne, University of Cologne, Cologne, Germany; 23 Department of Medical Statistics, University Medical Center Göttingen, Göttingen, Germany

**Keywords:** Randomized controlled trials, Molecularly targeted therapy

## Abstract

Ruxolitinib (RUX) is approved as second-line treatment for HU-intolerant/-resistant patients with polycythemia vera (PV). Here, we assessed RUX vs. best available therapy (BAT) as first-line treatment in PV in the randomized multicenter RuxoBEAT clinical trial (NCT02577926). Crossover from BAT to RUX was possible after 6 months. Baseline characteristics in the RUX (*n* = 44) and BAT (*n* = 34) arms were comparable. The primary endpoint, the clinico-hematologic complete response (CR) rate at month 6, was not met (2.3% vs. 2.9%, *p* = 1.000). Analyses of secondary and exploratory endpoints showed that RUX was significantly more effective than BAT in improving hematocrit, splenomegaly, and patient-reported pruritus and fatigue, but less effective in reducing platelet counts. Compared to baseline, JAK2V617F variant allele frequency decreased significantly in both RUX and BAT groups at 6 months. The time on treatment (*p* < 0.001) and duration of treatment (*p* = 0.022) were significantly superior for RUX vs. BAT during the 24-month study period. There were no new safety signals. These findings demonstrate that RUX did not lead to higher CR rates than BAT and suggest that RUX may induce clinically meaningful additional disease improvements in first-line therapy of PV patients.

## Introduction

Polycythemia vera (PV) is characterized by chronic trilineage expansion in the bone marrow and peripheral blood [1]. Patients with PV suffer from disease-related symptoms, including pruritus, headache, concentration problems, dizziness, and fatigue [2], and morbidity and mortality depend on the occurrence of vascular complications and progression to post-PV myelofibrosis (MF) or acute myeloid leukemia (AML) [1], thus rendering these important clinical endpoints to prevent. According to current guidelines, high-risk PV patients require cytoreductive therapy [1]. Hydroxyurea (HU) and peginterferon-alpha (pegIFNa) are approved in this setting [3]. However, most patients continue to suffer from PV-associated symptoms despite these treatments [4]. The JAK inhibitor ruxolitinib (RUX) is approved for HU-intolerant or -resistant PV [5, 6]. In the MAJIC-PV trial, RUX demonstrated improved event-free survival vs. BAT in these patients [7]. We hypothesized that RUX may have a higher efficacy than BAT in controlling the disease and its related symptoms as a first-line treatment in patients with PV.

A pre-specified futility analysis of the first RUX-treated PV patients had shown that first-line treatment of PV patients with RUX is feasible [8]. The present analysis reports the results from the randomized comparison of RUX vs. BAT treatment in the PV cohort after 6 months of therapy, and the 24-month results of patients continuously receiving RUX or BAT, or those that crossed over after 6 months from BAT to RUX (BAT-CO) treatment, as specified in the study protocol for patients meeting crossover criteria.

## Materials and methods

### Study design

RuxoBEAT is a German multicenter open-label two-arm phase 2b randomized controlled trial, assessing the efficacy and safety of RUX versus BAT in high-risk PV and essential thrombocythemia (ET) (Fig. S1). The results of the PV cohort are reported here.

The trial was approved by the central ethics committee at the site of the Coordinating Investigator (SK) at RWTH Aachen Faculty of Medicine (EK 345/14) and by the respective local ethics committee for each study site. The study was registered at clinicaltrials.gov (no. NCT02577926), EUCT (no. 2024-515619-23-00), and EudraCT (no. 2013-002132-25). All patients provided written informed consent before entering the study, and the study was conducted according to Good Clinical Practice Guidelines and the Declaration of Helsinki as an independent investigator-initiated and -sponsored trial, with RWTH Aachen University Hospital as the sponsor, and it was in part financially supported by Novartis. See supplementary information for a synopsis of the trial protocol.

### Participants

Eligible patients had to fulfill key inclusion criteria: ≥18 years of age; Eastern Cooperative Oncology Group performance status 0–2; WHO 2008 diagnostic criteria for PV; indication for cytoreduction defined by at least one of the following: age >60 years and/or previous documented thrombosis/thromboembolism and/or platelet count >1500 × 10^9^/l, poor tolerance of phlebotomy or frequent phlebotomy requirement and/or symptomatic or progressive splenomegaly and/or severe disease-related symptoms (according to the investigator´s definition) and/or progressive leukocytosis with leukocyte count >20 × 10^9^/l; never treated with cytoreductive drugs except HU, anagrelide, or interferon for up to 6 weeks maximum (phlebotomy and/or aspirin were allowed); adequate organ function. Key exclusion criteria included post PV-MF or post ET-MF (IWG-MRT); previous RUX treatment; inadequate bone marrow reserve (ANC ≤ 1 × 10^9^/l or platelet count <50 × 10^9^/l); active malignancy during the previous 3 years except treated cervical intraepithelial neoplasia, basal cell carcinoma of the skin, or squamous cell carcinoma of the skin, each with no evidence for recurrence in the past 3 years; any medical condition requiring prolonged use of >20 mg daily of oral corticosteroids (>1 month); active splanchnic vein thrombosis within the last 3 months.

All patients needed to have an indication for cytoreductive therapy to be enrolled. Patients were allowed a maximum of 6 weeks of PV-directed therapy prior to enrollment.

### Randomization and intervention

Patients were randomized in a 1:1 ratio to receive RUX or BAT, based on permuted blocks of varying lengths (concealed to the investigators and designed by the Institute of Medical Statistics and Computational Biology, Faculty of Medicine and University Hospital Cologne, University of Cologne, Cologne, Germany), stratified – for diagnosis ET, by age (<60, ≥60) and number of previous treatments (<2, ≥2); and for diagnosis PV, by age only (<60, ≥60). Due to a technical configuration error in the electronic Case Report Form (eCRF), treatment allocation was initially implemented without the planned stratification and blocking. This system error was identified and corrected in June 2019. Although this issue resulted in a numerical imbalance due to unblocked allocation and partially filled blocks, allocation concealment remained fully intact, precluding selection bias. The initial target population was 190 patients, each with PV or ET. Patients in the RUX arm received a starting dose of 10 mg twice daily (BID) and were allowed to dose-increase to up to 20 mg BID. BAT consisted of HU, pegIFNa, other therapy, or no cytoreductive therapy (Supplementary Fig. S3). Combination therapies were not allowed. Patients in the BAT arm who had to switch from the first to a second cytoreductive drug in the first 6 months with less than 140 days on the first treatment were considered as failures (none of the patients failed treatment between 140 and 168 days [between months 5 and 6]). Crossover of BAT patients to RUX was allowed after 6 months of BAT in cases of documented BAT intolerance and/or resistance (BAT-CO patients) (Fig. S1). Patients receiving RUX therapy (either from the beginning or after crossover) who benefitted from RUX treatment were allowed to receive RUX beyond 24 months until the end of the trial. See trial synopsis in the supplementary information for details on crossover eligibility and criteria for extended RUX medication beyond 24 months.

### Outcomes

The primary endpoint of the trial is the clinico-hematologic complete response (CR) rate, according to Barosi et al. [9], at month 6 (response criterion #1, see below). The Revised ELN criteria from 2013 [10] were not used since bone marrow histology was optional at month 6 in this study. To assess patients´ symptoms for the primary endpoint, we used a score of “zero” for the following patient-reported outcomes (PROMs): pruritus, headache, and the two symptoms of microvascular disturbances, dizziness and concentration problems (Likert scale of 0 to 10). PROMs were recorded using an MPN-SAF questionnaire [2] modified to include headache and quality of life parameters (courtesy of Mayo Clinic for academic use). Secondary endpoints included, among others, overall response (OR) rate, combining complete and partial responses (PR) [9], safety of both regimens, efficacy as assessed by both the absence of phlebotomy (hematocrit [HCT] <45%) and reduction in spleen size (palpable spleen that is reduced by >50% from baseline measured by palpation or, where available, ultrasound) at month 6 and month 24, and proportion of subjects achieving both durable absence of phlebotomy eligibility and durable spleen size reduction measured by palpation (PV) (durable defined as >3 months). Splenomegaly by ultrasound was defined as a spleen length of ≥13 cm. Exploratory endpoints included changes in relevant blood counts, spleen size reduction, and PROMs, as well as overall survival, event-free survival (EFS) (defined as absence of death, arterial thrombotic event, venous thrombotic event, serious hemorrhage, or transformation to MF or AML), and changes in *JAK2*V617F variant allele frequency (VAF, using genomic DNA from whole blood and subjected to next-generation sequencing, as described [11]). Crossover occurred after assessment of the primary endpoint parameters and was thus not a confounder for this endpoint.

### Statistical analysis

Statistical analysis is performed separately for PV and ET patients. In each of these strata, CR rates of 44% in the RUX arm and 24% in the BAT arm were assumed [12]. With a power of 80% and a two-sided significance level of *α* = 0.05, calculation of the sample size based on a group comparison using Pearson´s Chi-Squared Test resulted in 95 subjects per stratum in each treatment group required to be randomized (Fig. S1). Therefore, a total of 190 patients in the PV stratum were assumed. An adaptive interim analysis with an adjusted significance level of 0.005 was planned for the time when 95 PV patients were randomized. All further study analyses were conducted descriptively, with a *p* value of 0.05.

Recruitment had to be paused in 2020 during the COVID pandemic, and, therefore, the pre-specified interim analysis was prematurely conducted after 78 patients with PV had been randomized and had completed 6 months on study, and the preliminary 6-month results were reported in abstract form [13]. Recruitment of new patients was ended, as recommended by the independent data safety monitoring committee, after it had become clear from the interim analysis that the primary endpoint could not be reached, with a conditional power of 19.7%. Based on the planned significance level of 0.005 for this interim analysis, a power of 18.4% was calculated for *n* = 95 patients. This power decreased to 14.6% due to the reduced sample size of *n* = 77.

Patient characteristics are presented descriptively, with distributions of categorical variables reported as absolute and relative percentages and those of continuous variables reported as medians with interquartile ranges. The primary analysis was carried out as a modified intention-to-treat (mITT) analysis for endpoints up to 6 months, defined as all eligible and randomized patients who received at least one dose of study medication (*n* = 77). Endpoints at later time points were primarily analyzed in an as-treated analysis set, corresponding to the mITT set, but analyzing patients according to treatment received. Safety analysis was based on the safety analysis population, defined as all randomized and treated participants. Thus, results beyond 6 months of therapy were separately analyzed for RUX only, BAT only, and BAT-CO patients.

Binary endpoints, as the complete clinico-hematologic response, were analyzed using Fisher’s Exact Test or Pearson’s Chi-Squared Test comparing two independent treatment arms. For comparisons involving more than two independent groups, the Fisher–Freeman–Halton Exact Test was applied. The difference between two dependent time points for binary endpoints was analyzed using McNemar’s Test. Continuous and ordinal data were analyzed using the Mann–Whitney-*U*-Test for two independent treatment arms, Kruskal–Wallis Test for more than two independent treatment arms, and Wilcoxon Signed-Rank Test for comparison of different time points within one treatment group. Time-to-event endpoints were analyzed using Kaplan–Meier survival curves and compared using Log-Rank-Test.

For the assessment of time on treatment, duration of response, and event-free survival during the entire 24-month period of the trial, Cox regression was performed, with the two regular covariates, gender (male, reference: female) and age, as well as the time-dependent variable treatment group (BAT, reference: RUX). Here, events in the BAT-CO arm were counted in the group in which they occurred, thus, only the two treatments, BAT and RUX were compared.

The data cutoff date for this analysis was Dec 11th, 2024. Statistical analyses were performed using SPSS Statistics version 28.0.1.1 (IBM Corp., Armonk, NY, USA), IBM SPSS Statistics for Windows), and R version 4.4.0 [14].

## Results

### Baseline characteristics and patient disposition

Figure S2 shows the consort diagram. 84 patients were assigned to the PV stratum. After exclusion of 6 patients due to screening failure (7.1%), 78 patients were randomized to either RUX (*n* = 44) or BAT (*n* = 34 [mostly HU]) (Figs. S2 and S3). One patient never received RUX treatment, so the mITT population for the 6-month analysis consisted of 77 patients (RUX: *n* = 43; BAT: *n* = 34) (see Fig. S2). 17 (50%) patients in the BAT arm crossed over to RUX (termed BAT-CO below).

Baseline characteristics of patients in the RUX and BAT arms were comparable (Table 1). The most severe symptoms were fatigue and early satiety, followed by pruritus. Importantly, baseline overall quality of life was impaired to a clinically relevant degree (Table 1).

**Table 1:** Baseline demographics of PV patients in RuxoBEAT trial.

Variable	Total (*n* = 77)	RUX^$^ (*n* = 43)	BAT^$&^ (*n* = 34)	*p*
Median age [years] (min, max)	58 [32, 84]	58 [36, 84]	55.5 [32, 82]	0.133^a^
Female patients	29 (38%)	18 (42%)	11 (32%)	0.393^b^
Patients with ECOG 0 vs.1 vs. 2	52 (69%) vs. 22 (29%) vs. 1 (1%)	25 (60%) vs. 17 (41%) vs. 0 (0%)	27 (82%) vs. 5 (15%) vs. 1 (3%)	**0.036**^**b**^
Platelet count [1/nl]	490 [323, 680]	494 [323, 680]	489 [278, 687]	0.587^a^
White blood count [1/nl]	12.1 [8.1, 16.4]	13.5 [8.7, 17.1]	11.0 [8.1, 14.8]	0.168^a^
Hemoglobin*[g/dl]	13.8 [12.4, 14.9]	13.8 [12.2, 15.0]	14.0 [12.4, 14.8]	0.739^a^
Hematocrit*[%]	44.2 [42,47]	45.5 [42.4, 47]	43.8 [41.4, 48]	0.615^a^
Patients with splenomegaly	41 (53%)	23 (54%)	18 (53%)	0.971^b^
Pre-treated patients^§^ •Hydroxyurea •Anagrelide •(Peg)Interferon-alpha	25 (33%)	9 (21%)	16 (47%)	**0.015**^**b**^
	21 (28%)	9 (21%)	12 (36%)	
	0 (0%)	0 (0%)	0 (0%)	
	5 (7%)	0 (0%)	5 (15%)	
Median duration of previous therapy[days] (min, max)	23 [1,42]	24 [4,37]	22 [1,42]	0.550^a^
PROM Early satiety [points]	4 [0, 5]	4 [1,6]	2 [0, 5]	0.141^a^
PROM Abdominal discomfort [points]	1 [0, 4]	0 [0, 4]	2 [0, 4]	0.249^a^
PROM Fatigue/Inactivity [points]	5 [2,8]	4 [2,8]	5 [1,8]	0.831^a^
PROM Headache [points]	1 [0, 3]	1 [0, 3]	1 [0, 4]	0.278^a^
PROM Concentration problems (vs. before MPN) [points]	2 [0, 5]	2 [0, 5]	2 [0, 5]	0.973^a^
PROM Dizziness/Vertigo/ Lightheadedness [points]	2 [0, 5]	2 [0, 5]	2 [0, 6]	0.933^a^
PROM Numbness/Tingling (hands and feet) [points]	1 [0, 4]	1 [0, 5]	0 [0, 3]	0.201^a^
PROM Night sweats [points]	2 [0, 6]	2 [0, 6]	1.5 [0, 6]	0.738^a^
PROM Itching (Pruritus) [points]	3 [0, 7.5]	3 [0, 5]	4 [1,8]	0.290^a^
PROM Bone pain [points]	0 [0, 5]	0 [0, 5]	1 [0, 6]	0.483^a^
PROM Fever (>38.5 °C) [points]	0 [0, 0]	0 [0, 0]	0 [0, 0]	0.076^a^
PROM Weight loss (unintended in past 6 months) [points]	0 [0, 0]	0 [0, 0]	0 [0, 0]	0.713^a^
PROM Quality of Life (QoL) (0=best; 10=worst) [points]	5 [2,6]	5 [2,6]	5 [3,6]	0.767^a^

RUX: Ruxolitinib; ^&^BAT: best available therapy (consisting of hydroxyurea (75.9%), PegIFNa (10.3%), other chemotherapy or experimental therapy (6.9%), or no medical therapy (6.9%)); IQR: Interquartile range. p: *p* value for RUX vs. BAT. ^$^Assigned treatment arm. *Phlebotomies were allowed before baseline. ^§^Only up to 6 weeks of pre-treatment were allowed. PROM: Patient-reported outcome measures (Rating 0: none; 10=highest; except for QoL). Quantitative data are presented as mean ± standard deviation or median [Q25; Q75 (or range)] and qualitative data as number (frequency).

Significant *p* values are provided in bold.

^a^Mann–Whitney-U-Test.

^b^Pearson’s Chi-Squared Test.

Three patients treated with BAT (*n* = 3/34, 8.8%) terminated the trial within the first 6 months (see Fig. S2) due to withdrawal of consent, loss to follow-up, or family planning. Of the remaining patients, 15/74 patients (20.3%) terminated the trial between the end of month 6 [EOM6] and EOM24, with no significant difference between BAT (*n* = 4/14 28.6%), RUX (*n* = 8/43; 18.6%), and BAT-CO (*n* = 3/17; 17.6%), or RUX vs. BAT/BAT-CO combined (18.6% vs. 22.6%). Reasons for early termination by EOM24 are shown in Fig. S2. The adverse events leading to early termination were fatty liver disease with elevated liver enzymes, metastatic urothelial cancer, inflammation of the kidney and rectum, anemia, and acne rosacea.

Cytoreductive pretreatment prior to enrollment was allowed for up to 6 weeks. 33% of patients received such therapy (21% of RUX and 47% of BAT patients; *p* = 0.015) for a median duration of 23 days (range 1–42 days) (Table 1). Acetylsalicylic acid use was comparable (35/43 (81%) RUX and 29/34 (85%) BAT patients (*p* = 0.764)).

BAT consisted of HU (73.5%), pegIFNa (8.8%), other therapy (5.9%), or no cytoreductive therapy (11.8%). Median RUX dose was 10 mg BID at start and throughout the 24-month study period, showing that protocol-recommended dose increases for optimization of efficacy did not lead to an increase in the median dose. In the first 24 months, dose interruptions occurred in 12/43 (27.9%) RUX and 7/34 (20.6%) BAT/BAT-CO (combined) patients.

### Efficacy during the first six months of randomized therapy (RUX vs. BAT)

Patients in both treatment arms (RUX vs. BAT) showed significant improvements in disease-related signs and/or symptoms during the first 6 months of therapy vs. baseline (Table 2). including reduced median WBC and platelet counts (PLT), hematocrit (HCT), phlebotomy requirements, splenomegaly, and PROMs for early satiety, night sweats, pruritus, weight loss, and overall quality of life (but not the other PROMs tested) with RUX, while BAT significantly reduced only median platelet count, WBC, HCT, and phlebotomy requirements, but not spleen size or any of the tested PROM symptoms (Table 2).

**Table 2:** Responses at 6 months as compared to screening/baseline.

	RUX			BAT		
Time point	BL	M6	*p*	BL	M6	*p*
Platelets* [1/nl]	494 [323, 680]	386 [263, 526]	**<0.001**^a^	489 [278, 687]	263 [170, 502]	**<0.001**^a^
WBC [1/nl]	13.5 [8.1, 17.1]	8.8 [6.9, 13.4]	**<0.001**^a^	11.0 [8.1, 14.8]	6.6 [5.1, 11.2]	**0.017**^a^
Hgb [g/dl]	13.8 [12.2, 15.0]	13.2 [11.9, 14.3]	**0.022**^a^	14.0 [12.4., 14.8]	13.7 [12.6, 14.1]	0.243^a^
Hematocrit* [%]	45.5 [42.4, 47.0]	40.8 [35.6, 44.3]	**<0.001**^a^	43.8 [41.4, 48]	42.2 [40.1, 44.4]	**0.045**^a^
Pts with phlebotomy	40 (93%)	6 (14.3%)	**<0.001**^b^	29 (85.3%)	6 (19.4%)	**<0.001**^b^
Spleen size [cm]	15.4 [12.7, 19.0]	13.4 [10.9, 15.3]	**<0.001**^a^	14.5 [12.5, 18.8]	14.3 [12.3, 19.1]	0.377^a^
Pts with splenomegaly	23 (53.5%)	16 (37.2%)	**0.039**^b^	18 (52.9%)	15 (48.4%)	1.000^b^
PROM early satiety [points]	4 [1,6]	2 [0, 4]	**0.012**^**a**^	2 [0, 5]	2.5 [1,5]	0.818^a^
PROM Abdominal discomfort [points]	0 [0, 4]	0 [0, 2]	0.175^a^	2 [0, 4]	2 [0, 7]	0.634^a^
PROM Fatigue/Inactivity [points]	4 [2,8]	3 [1,7]	0.129^a^	5 [1,8]	5 [3,7]	0.284^a^
PROM headache [points]	1 [0, 3]	0 [0, 2]	0.722^a^	1 [0, 4]	1 [0, 6]	0.685^a^
PROM concentration pbs [points]	2 [0, 5]	2 [1,4]	0.629^a^	2 [0, 5]	3 [1,5]	0.397^a^
PROM dizziness/Vertigo/ Lightheadedness [points]	2 [0, 5]	2 [0, 5]	0.757^a^	2 [0, 6]	2 [0, 5]	0.977^a^
PROM Numbness/Tingling [points]	1 [0, 5]	1 [0, 5]	0.740^a^	0 [0, 3]	0 [0, 2]	0.846^a^
PROM Night Sweats [points]	2 [0, 6]	0 [0, 4]	**0.046**^**a**^	1.5 [0, 6]	1 [0, 4]	0.089^a^
PROM pruritus* [points]	3 [0, 5]	1 [0, 2]	**<0.001**^a^	4 [1,8]	4 [1,6]	0.211^a^
PROM Bone pain [points]	0 [0, 5]	0 [0, 2]	0.126^a^	1 [0, 6]	1 [0,4]	0.260^a^
PROM Fever (>38.5 °C) [points]	0 [0, 0]	0 [0, 0]	0.188^a^	0 [0, 0]	0 [0, 0]	1.000^a^
PROM Weight loss (unintended in past 6 months) [points]	0 [0, 0]	0 [0, 0]	**0.029**^**a**^	0 [0, 0]	0 [0, 0]	0.618^a^
PROM Quality of Life (QoL) (0=best; 10=worst) [points]	5 [2,6]	3 [1,5]	**0.034**^**a**^	5 [3,6]	4 [3,7]	0.512^a^

RUX: Ruxolitinib; BAT: best available therapy; BL: baseline; M6: month 6; *p*: *p* value; IQR: Interquartile range; WBC: white blood count; Hgb: hemoglobin; PROM: patient-reported outcome measures; pbs: problems. *p*: *p* value for BL vs. M6 (in addition, PROMs “early satiety” and “quality of life” were also significantly improved in the RUX arm).

Significant *p* values are provided in bold.

^*^p < 0.05 RUX M6 vs. BAT M6 (platelets, hematocrit, PROM pruritus); Quantitative data are presented as mean ± standard deviation or median [Q25; Q75] and qualitative data as number (frequency).

^a^Wilcoxon Signed-Rank Test.

^b^McNemar Test.

CR rate at month 6 was not significantly different between RUX vs. BAT (Table 3), and, therefore, the primary endpoint of the trial was not met. OR tended to be higher with RUX (*p* = 0.073) (Table 3). Hematologic response was, however, dependent on the response criteria used: #1 normal blood counts, absence of splenomegaly and PROM = 0, #2 same as #1 but less strict PROM of <3, #3 normal blood counts, absence of splenomegaly (by palpation or ultrasound) and of physician-assessed symptoms, #4 blood counts and absence of splenomegaly and physician-assessed pruritus, or #5 relying on response of blood counts and splenomegaly alone without symptom assessments (similar to the response criterion used in the MAJIC-PV study [7]). Indeed, with less stringent definitions of CR (#2 to #5), CR rates increased to over 20% (Table 3), while OR rates remained essentially unchanged, and differences for CR and OR remained non-significant between RUX and BAT treatment arms (Table 3).

**Table 3:** Efficacy at 6 and 24 months.

Response criterion EOM6	CR (%)				PR (%)				OR (CR + PR) (%)			
Treatment arm	RUX	BAT	N/A	*p*	RUX	BAT	N/A	*p*	RUX	BAT	N/A	*p*
#1 Barosi et al. [9] PROM = 0	2.3	2.9	N/A	1.000	79.1	58.8	N/A	N/A	81.4	61.8	N/A	0.073
#2 Barosi et al. [9] PROM < 3	11.6	5.9	N/A	0.455	72.1	61.8	N/A	N/A	83.7	67.6	N/A	0.112
#3 Splenomegaly and symptoms assessed by physician	14.0	14.7	N/A	1.000	67.4	50.0	N/A	N/A	81.4	64.7	N/A	0.120
#4 Splenomegaly and pruritus assessed by physician	16.3	14.7	N/A	1.000	67.4	50.0	N/A	N/A	83.7	64.7	N/A	0.067
#5 MAJIC-PV criteria (blood counts, spleen)	20.9	23.5	N/A	0.790	60.5	41.2	N/A	N/A	81.4	64.7	N/A	0.120
**Response criterion EOM24**	**CR (%)**				**PR (%)**				**OR (CR**+**PR) (%)**			
**Treatment Arm**	**RUX**	**BAT**	**BAT-CO**	***p***	**RUX**	**BAT**	**BAT-CO**	***p***	**RUX**	**BAT**	**BAT-CO**	***p***
#1 Barosi et al. [9] PROM = 0	2.3	0.0	0.0	1.000	69.8	61.5	76.5	N/A	72.1	61.5	76.5	0.674
#2 Barosi et al. [9] PROM < 3	9.3	7.7	0.0	0.567	69.8	61.5	76.5	N/A	79.1	69.2	76.5	0.746
#3 Splenomegaly and symptoms assessed by physician	20.9	30.8	5.9	0.219	62.8	38.5	70.6	N/A	83.7	69.2	76.5	0.445
#4 Splenomegaly and pruritus assessed by physician	27.9	29.4	11.8	0.427	58.1	23.5	64.7	N/A	86.0	69.2	76.5	0.320
#5 MAJIC-PV criteria (blood counts, spleen)	32.6	35.3	23.5	0.800	48.8	17.6	52.9	N/A	81.4	69.2	76.5	0.579

CR, PR, and overall response (OR) results at end of months 6 (top) and 24 (bottom), including alternative response criteria (see definitions in the manuscript). *P*-values (*p*) were calculated for comparisons between treatment groups for CR vs. no CR and for overall response vs. no overall response (Fisher’s Exact Test/ Fisher–Freeman–Halton Exact Test).

CR and PR response rates did not differ whether or not pretreatment (max. 6 weeks) was applied prior to baseline (Supplementary Fig. S3), nor with different types of BAT treatment during the 24-month treatment phase (Supplementary Fig. S4). Three BAT patients switched BAT treatment prior to crossover and were analyzed as failures.

We assessed potential reasons for failure to achieve CR and found that 14/43 (33%) of RUX and 5/34 (15%) of BAT patients did not have their dose increased by end of month 6 (EOM6), despite continuously elevated WBC, PLT, and/or HCT. However, dose increases were not mandatory, and the results showed that responses were not lower in patients who received lower mean doses of ruxolitinib (Fig. S8), suggesting that dose reductions did not lead to inferior responses. Also, during months 1–6 (EOM1-EOM6), the fraction of eligible patients in whom the RUX dose was escalated was the same whether toxicity was reported or not, suggesting that toxicity was not the limiting factor for the physician´s decision to dose escalate or not.

**Fig. 1:**
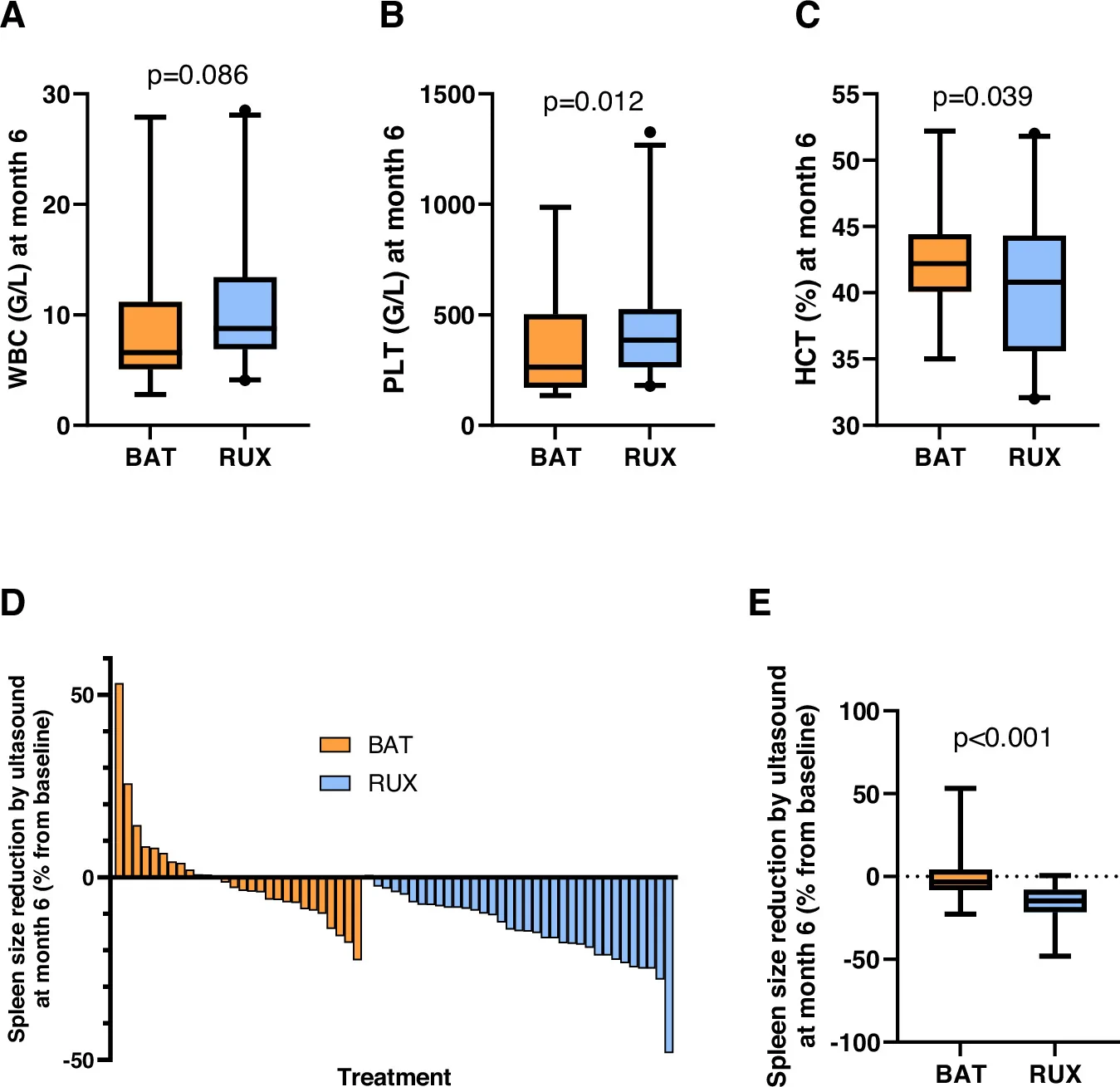
Responses at 6 months in the ITT population (BAT vs. RUX). **A** White blood cell counts (G/L) in both treatment arms. **B** Platelet counts (G/L) in both treatment arms. **C** Hematocrit (%) in both treatment arms. **D** Spleen size reduction at month 6 (% from baseline) in both treatment arms, depicted in a waterfall plot. **E** Spleen size reduction at month 6 (% from baseline) in both treatment arms, depicted in a box plot. **A**–**E** Boxplots display the median and interquartile range (IQR). Whiskers extend to the lowest and highest values within 1.5 times the IQR. Outliers are plotted as individual points.

At month 6, HCT control, defined as HCT below 45% and absence of phlebotomy, was achieved in 33/43 (76.7%) of RUX and 20/34 (58.8%) of BAT patients (*p* = 0.137). WBC were not significantly different in the two treatment arms (*p* = 0.086), PLT was significantly lower in the BAT arm (*p* = 0.012), and HCT was significantly lower in the RUX arm (*p* = 0.039) (Fig. 1C–E), and spleen size reduction, as assessed by abdominal ultrasound, more pronounced in the RUX arm (*p* < 0.001) (Fig. 1F, G). The percentage of patients who needed phlebotomies between months 3 and 6 was not different in the two treatment arms (*p* = 0.564, not shown).

**Fig. 2:**
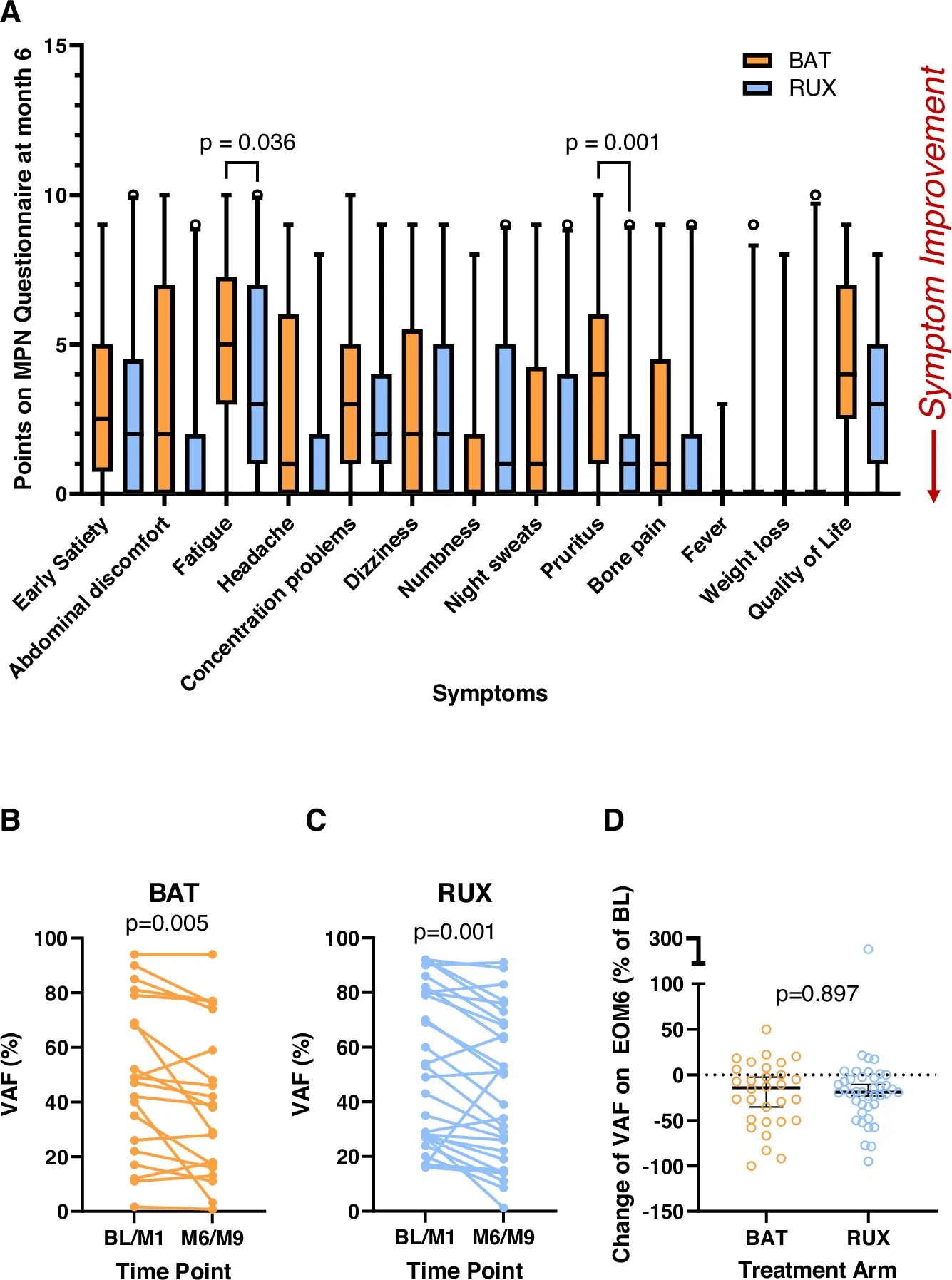
Symptom response and JAK2V617F variant allele fraction response at month 6. **A** Patient-reported outcome measures (PROM) for the depicted symptoms and for quality of life, each rated at a scale of 0–10, with decreasing points signifying improvement, in both treatment arms. Boxplots are shown with *p*-values from Kruskal–Wallis Tests. Symbols depict outliers. Variant allele frequency (VAF) of JAK2V617F-mutant DNA sequences in patients assigned to BAT (**B**) or RUX (**C**) treatment, depicted pairwise for the same patient at baseline (BL)/month1 (month 1 was used in rare cases when there was no data point for BL) vs. month 6/month9 (month 9 was used in rare cases when there was no data point for month 6). Mean VAF +/- standard deviation decreased in BAT-treated patients from 48.49+/- 28.43 at BL/M1 to 40.31+/- 27.99 at M6/M9 and in RUX-treated patients from 52.36+/- 27.73 at BL/M1 to 44.67+/- 27.19 at M6/M9. Pairwise Wilcoxon Signed-Rank Tests were performed. **D** VAF reduction at month6/month9 vs. BL/month1 was calculated for each treatment arm and compared using unpaired Mann–Whitney *U*-Tests.

Intriguingly, while there had been no significant differences in symptom severity at baseline, at month 6, patients in the RUX arm reported significantly lower symptom severities for pruritus (*p* = 0.001) and fatigue (*p* = 0.036) than the patients in the BAT arm (Fig. 2A, and Table 2). None of the symptoms showed improvements in the BAT vs. RUX arm at 6 months (Fig. 2A).

One of the exploratory endpoints was a reduction in JAK2V617F VAF. Both RUX and BAT were associated with a significant VAF reduction from baseline to month 6, with a median reduction of 18.5% and 12.9% (*p* < 0.001 and *p* = 0.009, respectively) (Fig. 2B, C), but without a significant difference in median VAF or VAF reduction between RUX- vs. BAT-treated patients (*p* = 0.559 and *p* = 0.752, respectively) (Fig. 2D).

### Safety during the first six months of therapy (RUX vs. BAT)

Safety analysis revealed 310 adverse events (AE) in both assigned treatment groups by month 6, including 158 AE in the RUX and 152 AE in the BAT group (11.4% and 8.6% grade ≥3, respectively). The proportion of AEs grade ≥3 of all AEs with RUX did not differ significantly from those with BAT (*p* = 0.452). When assessing safety profiles in individual patients (Table 4), 83% of patients experienced at least one adverse event, and 30% experienced a grade 3 or higher event. There were only minor differences in the two treatment arms, e.g., a lower percentage of gastrointestinal disorders in the RUX vs. BAT arm. Altogether, no new safety signals were detected in this analysis.

**Table 4:** Number of patients with adverse events (AE) during the first 6 months.

MEDDRA > System organ class & CTC-Grade < 3 vs. >=3	Total Count	% of (*n* = 77)	# Events	RUX Count	% of (*n* = 43)	# Events	BAT Count	% of (*n* = 34)	# Events	Difference A-B (%)	*p*-value^a^
ANY (S)AE	64	83.1	310	36	83.7	158	28	82.4	152	1.4	1.000
& CTC-Grade <3	41	53.2	279	23	53.5	140	18	52.9	139	0.5	.
& CTC-Grade >=3	23	29.9	31	13	30.2	18	10	29.4	13	0.8	.
MEDDRA	64	83.1	310	36	83.7	158	28	82.4	152	1.4	1.000
General disorders and administration site conditions	26	33.8	36	12	27.9	18	14	41.2	18	−13.3	0.237
& CTC-Grade <3	24	31.2	34	12	27.9	18	12	35.3	16	−7.4	.
& CTC-Grade >=3	2	2.6	2	.	0.0	.	2	5.9	2	−5.9	.
Eye disorders	4	5.2	7	3	7.0	6	1	2.9	1	4.0	0.626
& CTC-Grade <3	3	3.9	5	2	4.7	4	1	2.9	1	1.7	.
& CTC-Grade >=3	1	1.3	2	1	2.3	2	.	0.0	.	2.3	.
Surgical and medical procedures	1	1.3	1	1	2.3	1	.	0.0	.	2.3	1.000
& CTC-Grade <3	1	1.3	1	1	2.3	1	.	0.0	.	2.3	.
Respiratory, thoracic and mediastinal disorders	11	14.3	13	4	9.3	4	7	20.6	9	−11.3	0.199
& CTC-Grade <3	11	14.3	13	4	9.3	4	7	20.6	9	−11.3	.
Reproductive system and breast disorders	2	2.6	2	1	2.3	1	1	2.9	1	−0.6	1.000
& CTC-Grade <3	2	2.6	2	1	2.3	1	1	2.9	1	−0.6	.
Skin and subcutaneous tissue disorders	21	27.3	27	10	23.3	14	11	32.4	13	−9.1	0.444
& CTC-Grade <3	21	27.3	27	10	23.3	14	11	32.4	13	−9.1	.
Renal and urinary disorders	3	3.9	4	3	7.0	4	.	0.0	.	7.0	0.251
& CTC-Grade <3	2	2.6	3	2	4.7	3	.	0.0	.	4.7	.
& CTC-Grade >=3	1	1.3	1	1	2.3	1	.	0.0	.	2.3	.
Blood and lymphatic system disorders	6	7.8	10	2	4.7	2	4	11.8	8	−7.1	0.397
& CTC-Grade <3	6	7.8	10	2	4.7	2	4	11.8	8	−7.1	.
Gastrointestinal disorders	25	32.5	35	9	20.9	12	16	47.1	23	**−26.1**	**0.026**
& CTC-Grade <3	21	27.3	31	9	20.9	12	12	35.3	19	**−14.4**	.
& CTC-Grade >=3	4	5.2	4	.	0.0	.	4	11.8	4	**−11.8**	.
Nervous system disorders	28	36.4	39	15	34.9	21	13	38.2	18	−3.4	0.814
& CTC-Grade <3	26	33.8	37	15	34.9	21	11	32.4	16	2.5	.
& CTC-Grade >=3	2	2.6	2	.	0.0	.	2	5.9	2	−5.9	.
Ear and labyrinth disorders	2	2.6	2	1	2.3	1	1	2.9	1	−0.6	1.000
& CTC-Grade <3	2	2.6	2	1	2.3	1	1	2.9	1	−0.6	.
Vascular disorders	11	14.3	12	6	14.0	7	5	14.7	5	−0.8	1.000
& CTC-Grade <3	7	9.1	8	4	9.3	5	3	8.8	3	0.5	.
& CTC-Grade >=3	4	5.2	4	2	4.7	2	2	5.9	2	−1.2	.
Neoplasms benign, malignant and unspecified	1	1.3	1	1	2.3	1	.	0.0	.	2.3	1.000
& CTC-Grade >=3	1	1.3	1	1	2.3	1	.	0.0	.	2.3	.
Cardiac disorders	2	2.6	4	2	4.7	4	.	0.0	.	4.7	0.500
& CTC-Grade <3	1	1.3	3	1	2.3	3	.	0.0	.	2.3	.
& CTC-Grade >=3	1	1.3	1	1	2.3	1	.	0.0	.	2.3	.
Infections and infestations	20	26.0	27	8	18.6	10	12	35.3	17	−16.7	0.120
& CTC-Grade <3	18	23.4	24	6	14.0	7	12	35.3	17	−21.3	.
& CTC-Grade >=3	2	2.6	3	2	4.7	3	.	0.0	.	4.7	.
Psychiatric disorders	9	11.7	10	3	7.0	3	6	17.6	7	−10.7	0.172
& CTC-Grade <3	7	9.1	8	2	4.7	2	5	14.7	6	−10.1	.
& CTC-Grade >=3	2	2.6	2	1	2.3	1	1	2.9	1	−0.6	.
Musculoskeletal and connective tissue disorders	15	19.5	25	7	16.3	10	8	23.5	15	−7.3	0.564
& CTC-Grade <3	13	16.9	23	7	16.3	10	6	17.6	13	−1.4	.
& CTC-Grade >=3	2	2.6	2	.	0.0	.	2	5.9	2	−5.9	.
Metabolism and nutrition disorders	9	11.7	11	6	14.0	7	3	8.8	4	5.1	0.723
& CTC-Grade <3	9	11.7	11	6	14.0	7	3	8.8	4	5.1	.
Investigations	13	16.9	37	9	20.9	29	4	11.8	8	9.2	0.366
& CTC-Grade <3	9	11.7	31	5	11.6	23	4	11.8	8	−0.1	.
& CTC-Grade >=3	4	5.2	6	4	9.3	6	.	0.0	.	9.3	.
Injury, poisoning and procedural complications	7	9.1	7	3	7.0	3	4	11.8	4	−4.8	0.693
& CTC-Grade <3	6	7.8	6	2	4.7	2	4	11.8	4	−7.1	.
& CTC-Grade >=3	1	1.3	1	1	2.3	1	.	0.0	.	2.3	.

*CTC* common toxicity criteria, *AE* adverse event, *SAE* serious AE.

Significant *p* values are provided in bold.

^a^Fisher´s Exact Test RUX vs. BAT (without correction for multiple testing).

### Efficacy beyond six months of therapy (RUX vs. BAT vs. BAT-CO)

As stated above, 50% of patients in the BAT arm crossed over to RUX (BAT-CO cohort). Therefore, beyond 6 months of therapy, patients were analyzed in the as-treated analysis set. Responses at month 24 were analyzed with the same criteria as at month 6. Using these criteria, the CR and OR rates at month 24 were not different between RUX, BAT, and BAT-CO groups (Table 3), with CR rates over 30% and OR rates over 80% (Table 3), as well as best OR rates by month 24 of 41/43 (95.3%), 13/13 (100%), and 17/17(100%) of RUX, BAT, and BAT-CO patients, respectively (not shown).

At month 24, there was no significant difference in WBC, PLT, or HCT between BAT, BAT-CO, and RUX-treated patients (Fig. S6A–C), and no difference in phlebotomy requirement (*p* = 0.427). Spleen size reduction remained significantly more pronounced in RUX- vs. BAT-treated patients (Fig. S6D), while patients who crossed over from BAT to RUX showed an intermediate spleen response (Fig. S6D).

At month 24, overall PROM scores were comparable between the three treatment groups (BAT, BAT-CO, and RUX). However, between BL and 24 months, pruritus showed a significant intraindividual improvement at 24 months in the RUX and BAT-CO groups, but not in the BAT group (Fig. S7A–C) (BAT assessment was limited by missing data). Also, early satiety and weight loss were improved in the RUX group (*p* < 0.001), and night sweats were improved in the BAT-CO group (*p* = 0.004).

Median JAK2V617F VAF (%) at month 24 vs. baseline decreased significantly in the RUX (*p* < 0.001) but not BAT (*p* = 0.123) or BAT-CO (*p* = 0.129) groups (Fig. S7D). However, there was no significant difference in VAF or VAF reduction (%) at month 24 between RUX-, BAT-, or BAT-CO-treated patients (*p* = 0.938 [Kruskal–Wallis]).

No patient progressed to Post-PV-MF or AML during 24 months of therapy.

Patients deriving clinical benefit from RUX treatment were allowed to continue this treatment, and of RUX-receiving (RUX-only and RUX-after-crossover combined) patients, 47/60 (78%) still received continuing RUX at month 24 and 33/60 (55%) at the time of data cutoff. The fraction of patients continuing beyond month 24 was similar in the RUX-only (34/43) vs. RUX-after-crossover (11/17) patients (*p* = 0.324).

### Safety beyond six months of therapy (RUX vs. BAT vs. BAT-CO)

For assessment of safety beyond 6 months of therapy, patients were analyzed “as treated” according to the actual therapy they received over the course of 24 months, i.e., patients who continuously received BAT or RUX but also those who crossed over from BAT to RUX (BAT-CO) after 6 months. AE´s occurring during 24 months of therapy are displayed in Supplementary Table S5. The fraction of patients with AE´s differed significantly among the three treatment groups (*p* = 0.006), with a lower fraction in the BAT vs. BAT-CO or RUX group. However, when analyzing the data in more detail, the only MEDDRA system organ class that significantly differed among the three treatment groups was “blood and lymphatic system disorders” (*p* = 0.009), which was numerically more prevalent in the BAT-CO group than the BAT and RUX groups (see more details in Supplementary Table S5). Altogether, there were no new safety signals during the study duration of 24 months.

### Overall survival, duration of treatment and event-free survival

No patient died during the first 24 months of therapy. Thus, overall survival was not different between the treatment arms.

**Fig. 3:**
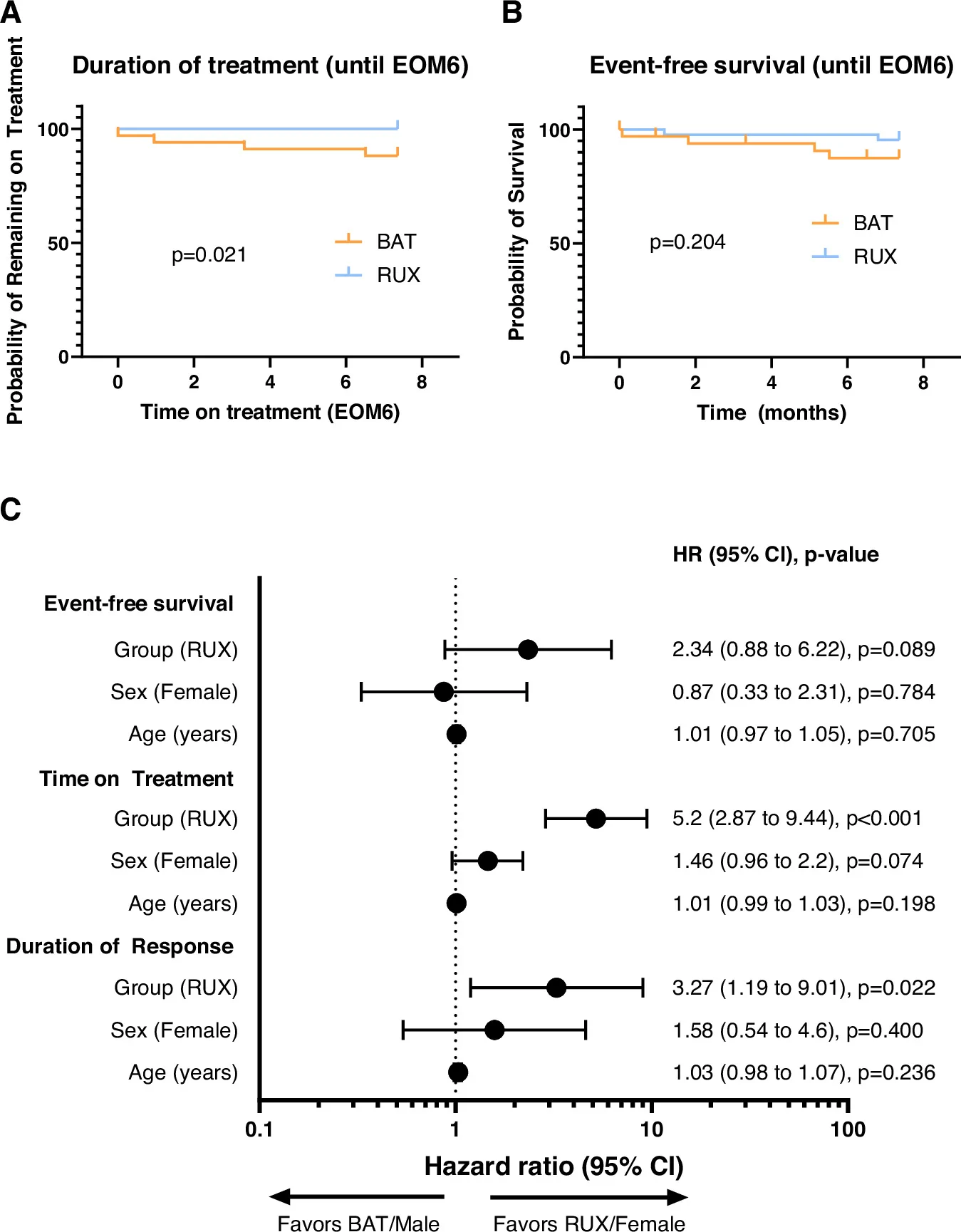
Duration of treatment, event-free survival, and time on treatment during the course of the 6- and 24-month study period. **A** Duration on treatment in the mITT population of assigned treatments within 6 months of therapy. Log-Rank Test was used to analyze for significant differences in BAT vs. RUX groups at 6 months. **B** Event-free survival (EFS) in the mITT population of assigned treatments within 6 months of therapy. EFS was defined as survival free of major thrombosis, major hemorrhage, transformation to myelofibrosis or AML, and death. Log-Rank Test was used to analyze for significant differences in BAT vs. RUX groups at 6 months. **C** Cox Regression analysis of EFS, time on treatment, and duration of treatment during the entire study period of 24 months.

During the first 6 months, time on treatment was significantly longer in RUX vs. BAT patients (Fig. 3A), while EFS was not significantly different between RUX and BAT (Fig. 3B).

However, Cox regression of the entire study period of 24 months revealed that time on treatment (HR 5.2 [CI 2.89, 9.44], *p* < 0.001) and duration of response (HR 3.27 [1.19, 9.01), *p* = 0.022) were significantly higher in patients receiving RUX than those receiving BAT (Fig. 3C). Also, EFS tended to be higher in patients on RUX receiving RUX than BAT (HR 2.34 [0.88, 6.22], *p* = 0.089) (Fig. 3C).

Major thrombosis, one of the most important disease complications, occurred in none of the RUX- vs. three of the BAT-treated patients in the first 6 months, and in three, two, and one patients, respectively, in the RUX, BAT, and BAT-CO treatment arms during months 6 to 24. When assessing single events of major thrombosis during 24 months of therapy, 7/9 events occurred in patients with abnormal counts around the time of the event, with 5/9 events associated with an elevated HCT (45% or more), 3/9 with elevated WBC, and 3/9 with elevated PLT counts. There were no episodes of non-major thrombosis during 24 months of therapy.

## Discussion

RuxoBEAT is a randomized trial of RUX vs. BAT in the first-line treatment of PV. JAK inhibition with RUX had been shown previously to decrease PV-associated HCT and phlebotomy requirement, splenomegaly, and symptoms in patients with HU-resistant or -intolerant PV [5, 6], leading to approval of RUX in this patient population.

In the randomized phase 3 RESPONSE trial (patients with HU-resistant/-intolerant PV who were phlebotomy-dependent and had splenomegaly), RUX was superior to standard-therapy (comprising 59% HU, 12% IFNa, 13% other, 15% no medication) in achieving the primary end point, defined as a combination of hematocrit control through week 32 and at least a 35% reduction in spleen volume at week 32, (21% RUX vs. 1% standard-therapy) [5], as well as secondary endpoints CR (24% vs. 9%, respectively) and total symptom score at week 32 [5]. AE´s were evenly distributed in both treatment arms, except for higher rates of herpes zoster infection with RUX (6.4%) vs. standard therapy (0%) [5]. Four RUX- vs. two standard-therapy-treated patients developed non-melanomatous skin cancer [5]. Comparable superiority for RUX was also documented in the randomized RESPONSE-2 trial, enrolling similar patients but without splenomegaly [6].

In the subsequent MAJIC-PV trial, again involving HU-resistant/-intolerant PV patients, CR after 12 months of therapy (43% vs. 26%, *p* = 0.02, modified ITT analysis) and duration of CR were superior with RUX vs. BAT [7]. Best OR at 12 months was not different between RUX (97%) and BAT (93%). Importantly, EFS with RUX was superior to BAT [7]. Safety analysis showed that infections (in particular, respiratory, genitourinary, and herpes zoster) were more frequent in the RUX vs. BAT patients (27 vs. 12 grade 3/4 events) and that squamous cell skin cancer was reported more frequently with RUX than BAT (11 vs. 0 events, respectively) [7].

While the primary endpoint of our trial was not met and percentages of CR after 6 months of therapy in the RUX and BAT arms (2.3% vs. 2.9% respectively) were lower than expected from previous data [12] used for our sample-size calculation, further analysis showed that the results of our RuxoBEAT trial in first-line PV were comparable to these three clinical trials in HU-resistant/-intolerant patients, when applying similar disease response criteria (Table 3). In particular, the omission of PROM points as response criteria yielded similar CR and OR rates (Table 3) as the previous trials, in which CR was defined simply by blood counts (RESPONSE [5]) or blood counts and spleen size (MAJIC-PV [7]). Even so, given that the MAJIC-PV patients were resistant or intolerant to HU, one could have expected a higher CR rate in the first-line PV patients treated in RuxoBEAT. One potential reason could be that doses were not increased in all RuxoBEAT patients (33% of RUX and 15% of BAT patients), leading to suboptimal responses. However, responses were not lower in patients who received lower mean doses of ruxolitinib (Fig. S8), suggesting that dose reductions did not lead to inferior responses. Neither was the median time from PV diagnosis to study treatment (378 days) (Fig. S9) longer than in MAJIC-PV (96 months) [7].

On the other hand, HCT control at month 6 in RuxoBEAT was achieved in 34/43 (79.1%) of RUX and 21/34 (61.8%) of BAT patients and was thus better than in the RESPONSE-2 trial [6], indicating that some disease parameters were better controlled in first- than second-line PV.

The discontinuation rate in our trial (3/78 [3.9%] at 6 months and 18/78 [23.4%] at 24 months) was comparable to the one in MAJIC-PV (23.9% at 12 months) [7]. Interestingly, a relevant fraction of RUX-receiving patients (78%) has continued RUX treatment long beyond 24 months, suggesting continued clinical benefit from this treatment.

Symptom assessment in RuxoBEAT showed an improvement in pruritus and fatigue after 6 months with RUX vs. BAT. Also, pruritus showed a significant intraindividual improvement at 24 months in the RUX and BAT-CO groups, suggesting a continuing beneficial effect of RUX on pruritus. However, when individual PROMs were compared among the three treatment groups (BAT, BAT-CO, RUX) at month 24, they did not differ significantly, possibly due to the splitting of the BAT group into BAT-CO and BAT patients, resulting in a smaller sample size, but also, given the high variance of symptoms at this time point, to a mixed symptom response, including improvements of some symptoms and worsening of others.

Safety analysis of RuxoBEAT showed comparable results to RESPONSE and MAJIC-PV. For example, the rate of grade 3/4 infections was 4.7% (RUX) and 0% (BAT) at 6 months and 7% (RUX), 0% (BAT), and 11.8% (BAT-CO) at 24 months in our trial. In the RESPONSE trial, these rates were 3.6% (RUX) and 2.7% (standard-therapy) at week 32 (month 8), and in MAJIC-PV, these rates were 23.7% (22/93 RUX patients) and 11.5% (10/87 BAT patients) [7]. No new safety signals were observed during the 24 months of this trial.

Our study has limitations. There was an imbalance between the two groups despite planned 1:1 randomization (44 versus 34), and although baseline characteristics were generally comparable, ECOG performance status differed between groups, and previous short-term cytoreductive therapy was significantly more frequent in BAT than RUX patients (47% versus 21%, *p* = 0.015). Although the randomization process was concealed from the investigators and these findings do not demonstrate selection bias, it is difficult to completely exclude it. Due to the allowed crossover from BAT to RUX after 6 months of therapy, the effects beyond this time point cannot be separated for the two treatments, and thus, the first 6 months represent the most informative randomized comparison period, while the subsequent as-treated analyses between months 6 and 24 were potentially affected by treatment selection and carry-over effects. Moreover, due to administrative reasons, recruitment was terminated during the COVID pandemic before the planned number of patients was reached, resulting in decreased power of the study, which may have affected both positive and negative findings. In addition, the inclusion of PROMs as clinical response criteria has generated uncertainty about when such PROMs are disease-related and which degree of PROMs correlates with physician-assessed symptoms used in previous PV trials. Such discrepancies between PROMs and physician-reported symptoms have been described after the RuxoBEAT trial had started [15]. Statistically, there was limited adjustment for multiple testing, and missing data were recorded as failures in the mITT analysis, which may potentially have underestimated the extent of the clinicohematologic response. Finally, BAT consisted primarily of hydroxyurea treatment, and may thus not reflect current routine clinical therapy since the approval and increasing use of (ro)pegylated interferon-alpha.

However, the strengths of RuxoBEAT remain. It is the first randomized analysis of RUX vs. BAT as first-line therapy in PV, with the same 24-month follow-up in both treatment arms. It shows that while CR rates at 6 months were not higher with RUX than BAT, RUX was more effective in controlling hematocrit and splenomegaly and alleviating PV-associated pruritus and fatigue, and that long-term RUX first-line treatment beyond 24 months is feasible and beneficial in patients with PV. Thus, PV patients with highly symptomatic disease and/or symptomatic splenomegaly may benefit from first-line RUX treatment.

We believe that our trial and recent trials, such as MAJIC-PV, provide sufficient evidence for further studies, e.g., a definitive phase III randomized trial of ruxolitinib vs. hydroxyurea or pegylated interferon-alpha as first-line therapy in PV.

## Supplementary information

Supplementary Material

Clinical Trial Synopsis

## Data Availability

The datasets and/or computer code generated during and/or analyzed during the current study are available from the corresponding author on reasonable request.
