# Supplementary material for: Firstline treatment with ruxolitinib versus best available therapy in patients with polycythemia vera: results from the randomized RuxoBEAT trial
Source: Blood Cancer J. 2026 Oct 22;16(1):189. doi: 10.1038/s41408-026-01644-2 (PMC13691084; doi:10.1038/s41408-026-01644-2)
Supplement: Supplementary file 1 [file 41408_2026_1644_MOESM2_ESM.pdf]

Ruxolitinib versus Best Available Therapy in patients with high-risk polycythemia vera or high-risk essential thrombocythemia – The Ruxo-BEAT Trial

Study protocol

12-181

This protocol is an integration of the following documents:

- Initial Protocol, Version 1.0
- Protocol Amendment No. 4 – integrated Protocol Version 5.0 (Date 26.09.2023)

CONFIDENTIAL

**Sponsor:**	University Hospital Aachen AöR,<br>represented by the Managing Board,<br>represented by the Dean of the Medical Faculty of the RWTH Aachen University<br>Prof. Dr. rer. nat. Stefan Uhlig
**Coord. Investig.:**	Prof. Dr. med. Steffen Koschmieder (LKP)
**EudraCT Number:**	2013-002132-25
**NCT:**	NCT02577926
**Protocol Code:**	12-181
**Running Title:**	Ruxo-BEAT

For the Sponsor: The Dean of the Medical Faculty of the RWTH Aachen University Prof. Dr. rer. nat. Stefan Uhlig
Pauwelsstraße 30, 52074 Aachen, Germany

Coordinating Investigator (LKP): Prof. Dr. med. Steffen Koschmieder
Department of Internal Medicine (Oncology, Hematology, Hemostaseology and Stem Cell Transplantation)
University Hospital RWTH Aachen
Pauwelsstr. 30, 52074 Aachen, Germany

Co- Coordinating Investigator: Prof. Dr. med. Tim. H. Brümmendorf
Department of Internal Medicine (Oncology, Hematology, Hemostaseology and Stem Cell Transplantation)
University Hospital RWTH Aachen
Pauwelsstr. 30, 52074 Aachen, Germany

Trial Statisticians: Prof. Dr. rer. medic. Martin Hellmich
Julia Frank, M.Sc.
Institute of Medical Statistics and Computational Biology
Medical Faculty and University Hospital Cologne
University of Cologne
Kerpener Str. 62, 50937 Koeln, Germany

1 STUDY SYNOPSIS

**EudraCT No.**	2013-002132-25
**Sponsor:**	University Hospital Aachen AöR
**Title:**	Ruxolitinib versus Best Available Therapy in patients with high-risk polycythemia vera or high-risk essential thrombocythemia
**Short Title:**	Ruxo-BEAT Trial
**Coordinating Investigator**	Prof. Dr. med. S. Koschmieder (LKP)
**Co-Coordinating Investigator**	Prof. Dr. med. T. H. Brümmendorf
**Study Diagram (R=Randomization):**	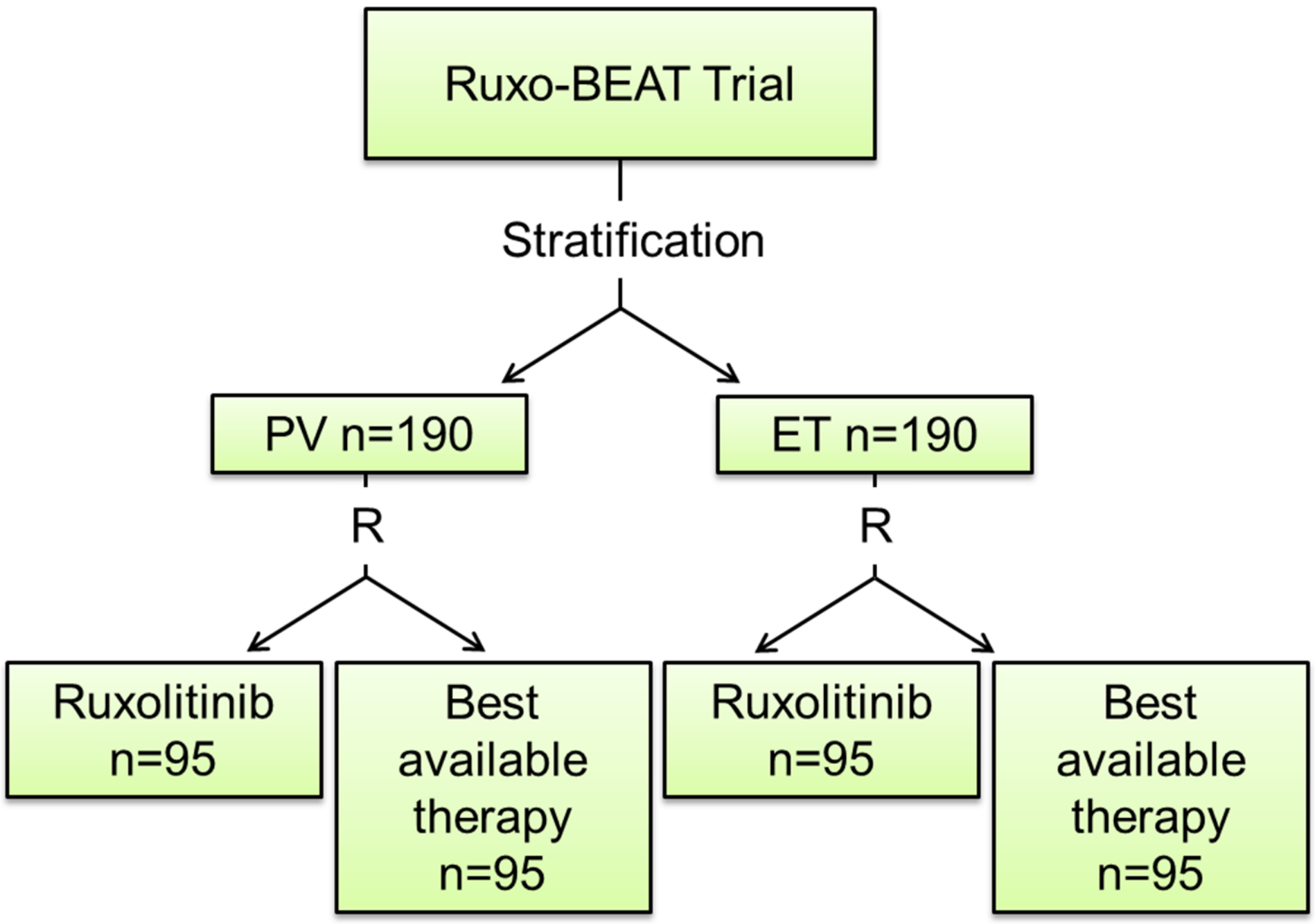
**Stratification**	polycythemia vera (PV) vs. essential thrombocythemia (ET)
**Randomization**	Ruxolitinib vs. best available therapy (monotherapy with i.e. hydroxyurea, anagrelide, PEG-interferon, busulfan etc. or best supportive care)
**Rationale**	The Philadelphia chromosome negative myeloproliferative neoplasms (MPN) comprise a group of clonal hematological malignancies that are characterized by chronic myeloproliferation, splenomegaly, different degrees of bone marrow fibrosis, and disease-related symptoms including pruritus, night sweats, fever, weight loss, cachexia, and diarrhea. In addition, due to elevated numbers of leucocytes, erythrocytes and/or platelets, the disease course can be complicated

	by thromboembolic disease, hemorrhage, and leukemic transformation as well as myelofibrosis.<br>Patients with polycythemia vera (PV) typically harbor an increased number of blood cells from all three hematopoietic cell lineages due to clonal amplification of hematopoetic stem cells, while patients with essential thrombocythemia (ET) typically show a predominant expansion of the megakaryocytic lineage. Most patients with PV below the age of 60 years are currently being treated with acetylsalicylic acid +/- phlebotomy only, and patients with low-risk ET have an almost normal life expectancy and often do not require specific treatment. However, PV- as well as ET-patients with a higher risk for complications require cytoreductive treatment (Lengfelder, et al., 2010). In addition, constitutional symptoms can be unbearable to patients even in the absence of bona fide high risk factors, and these patients may similarly benefit from antineoplastic therapy.<br><br>Initial results of the interim analysis showed that it is statistically highly unlikely to reach the primary endpoint. Therefore, following the recommendation of the DMC, recruitment was terminated early. However, as there are no safety concerns for the trial and longterm scientific results continue to be important, active patients will continue to be treated according to the current protocol until the planned end of the Ruxo-BEAT trial.
**Study Objective and Endpoints**	The objective of the Ruxo-BEAT trial is to assess the feasibility, safety, and efficacy of administration of ruxolitinib vs. best available therapy in patients with high-risk PV or high-risk ET.<br><br>**Primary Endpoint**<br>• The rate of complete clinicohematologic responses (CHR) at month 6, compared to baseline, as defined by (Barosi, et al., 2009). For a detailed definition of response criteria and assessment of the primary endpoint, see section 15.5.<br>**Secondary Endpoints**<br>• Safety of both regimens<br>• The rate of complete clinicohematologic responses (CHR) at month 12 and month 24 as defined by Barosi *et al.* Blood 2009 (with the clinical modification that splenomegaly assessment may be done by ultrasound or palpation)<br>• To determine the complete response (CR) rate at month 6 as defined by Barosi *et al*. Blood 2013 (revised ELN response criteria, see section 15.6) (Barosi, et al., 2013)<br>• The efficacy as assessed by both the absence of phlebotomy (Hct <45%) and reduction in spleen size (palpable spleen that is reduced by > 50% from

baseline measured by palpation or ultrasound) OR platelet count < 600 x 10^9/l (ET) at month 6 and month 24

- The proportion of subjects achieving both durable absence of phlebotomy eligibility and durable spleen size reduction measured by palpation (PV) OR durable platelet count < 600 x 10^9/l (ET) (durable defined as > 3 months) (Barosi *et al*. 2009/2013) at month 6, month 12 and month 24
- The rate of overall clinicohematologic responses (CR + PR) according to both guidelines (Barosi *et al*. 2009 and 2013) at month 6 and month 24

Exploratory Endpoints

- Overall survival
- Progression free survival (see section 7.16 for definition of disease progression)
- Complication free survival (defined as absence of death, arterial thrombotic event, venous thrombotic event, and serious hemorrhage)
- Event free survival (defined as absence of death, arterial thrombotic event, venous thrombotic event, serious hemorrhage, transformation to myelofibrosis, acute myeloid leukemia, myelodysplasia, and progression)
- Rate of durable complete hematologic remission (defined as platelet count ≤ 400 x 10^9/l, white blood cell count ≤ 10 x 10^9/l AND hematocrit < 45% without phlebotomy for more than 3 months) at month 6 and month 24
- Rate of durable phlebotomy independence (defined as phlebotomy independence for more than 3 months with an hematocrit < 45%) at month 6 and month 24
- To estimate the duration of the overall clinicohematologic response (as defined by Barosi *et al.* 2009 vs. Barosi *et al.* 2013)
- Patient-reported outcome and symptoms (measured by survey)
- To compare the proportion of subjects achieving normalization of white blood cell counts at month 6 and month 24
- To compare the proportion of subjects achieving a reduction of platelet counts to defined levels at month 6 and month 24
- Sex-specific differences
- Preliminary efficacy assessment in the PV arm after 6 months of therapy (n=50 patients [randomized to either ruxolitinib or BAT]). This will be conducted as a futility analysis with stopping if no favorable trend (as judged by the DMC) is observed for ruxolitinib for any of the following criteria:
    - Improvement in the hematocrit level during 6 months of treatment
    - Improvement of one of the following 3 symptom parameters assessed by physician´s judgement or via MPN-SAF during 6 months of treatment:

	▪ Pruritus<br>▪ Night sweats<br>▪ Bone pain<br>• Complete clinicohematologic response rate (CHR) by month 6, 12, 24 compared to baseline as defined by (Barosi, et al., 2009)
**Inclusion Criteria**	1. Subjects must provide written informed consent prior to study-specific procedures or assessments which are not routinely performed for diagnosis or monitoring of PV or ET, and the subjects must be willing to comply with treatment and to follow up assessments and procedures<br>2. Patient must be 18 years of age or older<br>3. Patient´s ECOG performance status must be 0-2<br>4. Patient must fulfill WHO 2008 diagnostic criteria for either polycythemia vera (PV) or essential thrombocythemia (ET). Moreover, PV- and ET-patients have to be classified as high risk according to defined criteria as outlined below.<br>- For patients with **high risk PV** OR **PV with indication for cytoreductive therapy due to progressive myeloproliferation**, AT LEAST ONE of the following must be fulfilled (according to DGHO onkopedia) (Barbui, et al., 2011), (Passamonti, 2009):<br>• Age > 60 years<br>• Previous documented thrombosis or thromboembolism<br>• Platelet count > 1500 x 10^9/l<br>• Poor tolerance of phlebotomy or frequent phlebotomy requirement<br>• Symptomatic or progressive splenomegaly<br>• Severe disease-related symptoms (according to the investigators definition)<br>• Progressive leukocytosis with leukocyte count > 20 x 10^9/l<br>- For patients with **high risk ET**, AT LEAST ONE of the following must be fulfilled (according to DGHO guidelines):<br>• Age > 60 years<br>• Platelet count > 1500 x 10^9/l<br>• Previous thrombosis or thromboembolism<br>• Previous severe hemorrhage related to ET (defined as decrease of Hgb of at least 2 g/dl)<br>5. Patients must fulfill the following criteria regarding prior therapy:<br>**PV patients**:

	Never treated with cytoreductive drugs except hydroxyurea, anagrelide, or interferon for up to 6 weeks maximum (phlebotomy and/or aspirin are allowed)<br>**ET patients:**<br>Naïve and pretreated patients may be entered in this trial.<br>6. Patient must have adequate liver function as indicated by a total bilirubin, AST, and ALT ≤ 2 of the institutional upper limit of normal (ULN) value, unless directly attributable to the patient's MPN<br>7. Patient must have a creatinine clearance >40ml/min calculated according to the modified formula of Cockcroft and Gault, eGFR, or directly measured after 24h-urine collection<br>8. Patient must be able to swallow and retain oral medication
**Exclusion Criteria**	1. Patients who meet criteria for post PV-MF or post ET-MF (IWG-MRT)<br>2. Patients who have received previous ruxolitinib treatment<br>3. Patients who have a history of anaphylaxis following exposure to the BAT drug of choice<br>4. Patients who have an inadequate bone marrow reserve as demonstrated by ANC ≤ 1 x 10^9/l OR platelet count <50 x 10^9/l<br>5. Patients who have known hepatitis B or C or HIV infection<br>6. Patients who suffer from other severe, concurrent diseases, including tuberculosis, serious cardiac functional dysfunction (class III or IV as defined by the New York Heart Association Classification), uncontrolled diabetes, uncontrolled hypertension, severe pulmonary disease (i.e. COPD with hypoxemia), or major organ malfunction that could interfere with the patient's ability to participate in the study<br>7. Patients who have history of active substance or alcohol abuse within the last year<br>8. Female patients who are pregnant or nursing<br>9. Patients who have participated in another interventional trial and/or used investigational agents or concurrent anticancer treatment for concomitant disease within the last 4 weeks of registration<br>10. Any circumstance at the time of study entry that would preclude completion of the study or the required follow-up prohibits inclusion into this study<br>11. Subjects who have had an active malignancy during the previous 3 years except for treated cervical intraepithelial neoplasia, basal cell carcinoma of the skin, or squamous cell carcinoma of the skin, each with no evidence for recurrence in the past 3 years<br>12. Patients who have uncontrolled bacterial, viral, or fungal infection<br>13. Patients who have any medical condition requiring prolonged use of oral corticosteroids with a dose of more than 20 mg per day (> 1 month)<br>14. Patients who have severe cerebral dysfunction and/or legal incapacity

	15. Patients who have had active splanchnic vein thrombosis within the last 3 months (includes Budd-Chiari, portal vein, splenic and mesenteric thrombosis)<br>16. Patients who have thyroid dysfunction which is not adequately controlled<br>17. Fertile men or women of childbearing potential cannot be included unless they are:<br>-surgically sterile or > 2 years after the onset of menopause and/or<br>-willing to use a highly effective contraceptive method (Pearl Index <1) such as oral contraceptives, intrauterine device, sexual abstinence, or barrier method of contraception (i.e. condoms) in conjunction with spermicidal jelly during study treatment<br>18. Patients who are taking any of the following prohibited medication:<br>-clarithromycin, telithromycin, troleandomycin (antibiotics)<br>-ritonavir, indinavir, saquinavir, nelfinavir, amprenavir, lopinavir (HIV protease inhibitors)<br>-itraconazole, ketoconazole, voriconazole, fluconazole (antifungals)<br>19. Patients with a diagnosis of galactose or lactose intolerance or a glucose-galactose-malabsorption
**Study Design**	This is an open-label, controlled, multi-center phase IIB clinical trial in which patients with polycythemia vera (PV) or essential thrombocythemia (ET) will be stratified by diagnosis and then randomized to one of the following two treatment arms:<br>1. Ruxolitinib **(experimental arm)**<br>2. Best available therapy (BAT) **(standard therapy)**<br>Ruxolitinib will be administered orally at a dose of 10 mg twice daily (both PV and ET), and BAT may include all currently used treatment options. BAT is the investigator's choice (monotherapy with i.e. hydroxyurea, anagrelide, interferon, busulfan, immunomodulators etc. or best supportive care). After providing written informed consent, eligible patients will be screened, then, if data is sufficient and inclusion and no exclusion criteria are met, will be randomized and start therapy on day 1 of the study. Patients in Arm A (Ruxolitinib) and patients after cross-over to ruxolitinib will be treated within the study as long as they benefit from the treatment. Clinical benefit will be defined as follows:<br>Patients with at least a stable disease + no signs of disease progression + any of the following items:<br>• Durable (lasting at least 6 months) improvement in any symptom of at least 2 points in comparison to baseline assessment as measured by the MPN-SAF-TSS<br>• Improvement in spleen size of at least 10% in comparison to baseline assessment

	• Absence of phlebotomy with durable hematocrit levels < 45% (for PV patients only)<br>• Improvement of platelet count < 800 10^9/l if at least two other drugs did not achieve this goal (for ET patients only)<br>• Documentation of other significant improvements as assessed by the local investigator and approved by the coordinating investigator.<br><br>Stable disease is defined as the absence of CR, PR or disease progression.<br><br>Patients who remain in arm B complete the treatment schedule 24 months and 12 months of follow-up within the trial. This patient cohort will afterwards be followed up within the GSG-MPN registry. Furthermore, for patients failing BAT at the 6 month time point, cross-over is possible (see section 7.17 for cross-over requirements).<br><br>Throughout the study, peripheral blood and BM assessments will be carried out as specified in detail in the protocol. Treatment will be continued in both arms until one of the following events occur: disease progression (includes death due to disease progression), any AE (includes death not due to disease progression) which in the opinion of the investigator precludes the continuation of study participation or places the patient at an intolerable risk, protocol deviation (as determined by the local principal investigator), termination of the study, patient being lost to follow-up, investigator discretion (reason to be specified), or decision by subject.<br><br>In addition to laboratory and clinical data, patient-reported outcome measures (QoL) in each treatment arm will be assessed at certain time points using the MPN-SAF TSS questionnaire.
**Study Duration**	Start of recruitment (FPFV): q4/2015<br>End of recruitment (LPFV): 08/2020<br>Study treatment duration:<br>**Arm A and patients after cross-over to Arm A:** patients with benefit (defined as CR or PR, or stable disease with clinical benefit as described in the section 5.8 “Study Design”) from treatment may continue therapy until progression, unacceptable toxicity or withdrawal. Afterwards, these patients will be followed up within the trial (see section 6.9) and after that via the GSG-MPN-registry.<br>**Arm B:** Maximum 36 months (24 months active treatment phase + 12 months of FU within the study); afterwards successful treatment should be continued until disease progression or intolerance, this will be followed up within the GSG-MPN registry.<br>In q4/2024, the study duration will be reassessed based on clinical data available at that point of time in terms of a risk-benefit-assessment.

	Study end (LPLV): anticipated q4/2027<br>Database Lock: anticipated q2/2028<br>Completion of Clinical Study Report (CSR): anticipated q4/2028
**Number of Patients**	The statistical analysis will be performed separately for the two different entities. For the chosen primary endpoint, we assumed that at least 44% of subjects in the ruxolitinib group and no more than 24% in the control (best-available therapy) group would achieve a complete clinico-hematological response. Under this assumption, a sample size 2 x 95 subjects for each diagnosis group would provide a power of at least 80% at a two-sided level of 0.05 using the chi-square test. An adaptive interim analysis will be performed after 95 patients, who have been treated for at least 6 month, with an adjusted significance level of 0.005. If recruiting in one or both strata is stopped before 95 patients are evaluable for the primary endpoint, the interim analysis is carried out for the evaluable patients.<br><br>Initially, it was planned that 380 patients (190 in each diagnosis stratum and 95 in each randomization arm) would be enrolled in the study, with a 2:1 ratio of women and men (ET; (Brière, 2007)) and 2:3 (PV; (McNally, et al., 1997)), respectively. However, based on the DMC's recommendation, recruitment was stopped prematurely after enrolment of 223 patients and randomization of 207 patients, as the primary endpoint is unlikely be met statistically.
**Treatment under study**	Patients will be treated until progression, unacceptable toxicity or withdrawal of consent. Those not achieving at least a confirmed PR at 6 months may be considered for other treatment options but may also continue with study treatment according to the treating physician´s decision or, if they were in the BAT arm of the study, will be assessed for cross-over eligibility.
**Safety Assessments**	Samples for hematology and serum chemistry will be analyzed locally. All subjects will have samples of blood collected on a regular basis for analysis of serum chemistry and hematology.
**Efficacy Parameters**	The antitumor activity will be assessed every 3 months during the first 12 months, then every 6 months thereafter, based on clinical and laboratory parameters. At defined time points, bone marrow assessments and ultrasound will also be performed for efficacy analysis. The primary efficacy endpoint is the complete clinicohematologic response rate (for detailed definition see section 15.5).<br><br>Patients will be withdrawn for disease progression, withdrawal of consent, or unacceptable toxicity.<br>Response will be assessed using clinical examination, blood tests, bone marrow examination if available.
**Study Drug Supplies**	Ruxolitinib is not approved for the use in patients with PV (except for patients with PV with hydroxyurea intolerance or resistance) or ET and will be supplied by

	Novartis during the trial. If ruxolitinib is approved in the included entities during the trial, commercially available preparations of this drug will be used.
