# Supplementary material for: Firstline treatment with ruxolitinib versus best available therapy in patients with polycythemia vera: results from the randomized RuxoBEAT trial
Source: Blood Cancer J. 2026 Oct 22;16(1):189. doi: 10.1038/s41408-026-01644-2 (PMC13691084; doi:10.1038/s41408-026-01644-2)
Supplement: Supplementary file 2 [file 41408_2026_1644_MOESM1_ESM.docx]

**SUPPLEMENTARY MATERIAL**

**Firstline treatment with ruxolitinib versus best available therapy in patients with polycythemia vera: results from the randomized RuxoBEAT trial**

Running Title: Firstline ruxolitinib vs. BAT in polycythemia vera

Steffen Koschmieder^1,2,3,*^, Susanne Isfort^1,2,3,4^, Lino L. Teichmann^2,5^, Philippe Schafhausen^6^, Florian H. Heidel^4,7^, Carl Crodel^7^, Andreas Hochhaus^7^, Martin Griesshammer^8^, Katja Sockel^9^, Philipp J. Jost^10,11^, Denise Walther^12^, Konstanze Döhner^13^, Joachim R. Göthert^14^, Markus Radsak^15^, Marinela Augustin^16^, Heiko Becker^17^, Aristoteles Giagounidis^18^, Markus Schaich^19^, Martine Klausmann^20^, Nikolas von Bubnoff^21^, Frank Stegelmann^13^, Maike Kortmann^2,22^, Michelle Zentis^2,22^, Julia Frank^23^, Anna Hagemeier^23^, Martin Hellmich^23,24^, Tim H. Brümmendorf^1,2^, for the German Study Group for Myeloproliferative Neoplasms (GSG-MPN)

^1^Department of Medicine (Hematology, Oncology, Hemostaseology, and SCT), Faculty of Medicine, RWTH Aachen University, Aachen, Germany

^2^Center for Integrated Oncology Aachen Bonn Cologne Düsseldorf (CIO ABCD), Aachen, Germany

^3^These authors contributed equally

^4^Hematology, Hemostasis, Oncology and Stem Cell Transplantation, Hannover Medical School (MHH), Hannover, Germany

^5^Department of Medicine III, University Hospital Bonn, Bonn, Germany

^6^Department of Oncology, Hematology, and Bone Marrow Transplantation with Section of Pneumology, University Medical Center Hamburg-Eppendorf, Hamburg, Germany

^7^Clinic for Internal Medicine 2, Jena University Hospital, Jena, Germany

^8^University Clinic for Haematology, Oncology, Haemostaseology and Palliative Care, Johannes Wesling Medical Centre Minden, University of Bochum, Minden, Germany

^9^Medical Clinic and Policlinic I, University Hospital Dresden, TU Dresden, Germany

^10^Medical Department III, Hematology and Oncology, and Center for Translational Cancer Research, Translatum, Technical University of Munich, Germany

^11^Division of Clinical Oncology, Internal Medicine, Medical University of Graz, Auenbruggerplatz 15, 8036 Graz, Austria

^12^Department of Hematology/Oncology and Celltherapy, Otto-von-Guericke University Magdeburg, Medical Center, Magdeburg, Germany

^13^Department of Internal Medicine III, University Hospital of Ulm, Ulm, Germany

^14^Department of Hematology and Stem Cell Transplantation, West German Cancer Center, University Hospital Essen, Essen, Germany

^15^UCT Mainz, Johannes Gutenberg University Mainz, Mainz, Germany; Department of Hematology and Medical Oncology, University Medical Center, Mainz, Germany; DKTK, Mainz, Germany

^16^Klinikum Nürnberg Nord, Klinik für Innere Medizin 5, Schwerpunkt Onkologie/Hämatologie, Paracelsus Medizinische Privatuniversität, Campus Nürnberg, Germany

^17^Department of Hematology, Oncology and Stem Cell Transplantation, Medical Center, Faculty of Medicine, University of Freiburg, Freiburg, Germany, and German Cancer Consortium (DKTK), partner site Freiburg, Germany

^18^Dept. Oncology, Hematology and Palliative Care, Marienhospital Düsseldorf, Düsseldorf, Germany

^19^Department of Hematology, Oncology and Palliative Medicine, Rems-Murr-Klinikum, Winnenden, Germany

^20^Gemeinschaftspraxis, Aschaffenburg, Aschaffenburg, Germany

^21^Department of Hematology and Oncology, University Medical Center Schleswig-Holstein and University Cancer Center Schleswig-Holstein (UCCSH), University of Lübeck, Lübeck, Germany

^22^Center for Translational & Clinical Research (CTC-A), Faculty of Medicine, RWTH Aachen University, Aachen, Germany

^23^Institute of Medical Statistics and Computational Biology, Faculty of Medicine and University Hospital Cologne, University of Cologne, Cologne, Germany

^24^University Medical Center Göttingen, Department of Medical Statistics, Göttingen, Germany

***Corresponding author:**

Prof. Dr. med. Steffen Koschmieder, Department of Medicine (Hematology, Oncology, Hemostaseology, and Stem Cell Transplantation), Faculty of Medicine, RWTH Aachen University, 52074 Aachen, Germany. E-mail: [skoschmieder@ukaachen.de](mailto:skoschmieder@ukaachen.de)

**Supplementary Figure 1 (Fig. S1):** Trial Design of the RuxoBEAT study.

**Supplementary Figure 2 (Fig. S2):** Consort diagram of PV patient numbers and distribution.

**Supplementary Figure 3 (Fig. S3):** Responses in patients at 6 months according to pretreatment (up to 6 weeks prior to baseline).

| **Pretreatment** | **RUX** | | | | **BAT** | | | |
| --- | --- | --- | --- | --- | --- | --- | --- | --- |
| *Strict response criteria #1 (symptom threshold = 0)* | NR (n) | PR (n) | CR (n) | **Total (n)** | NR (n) | PR (n) | CR (n) | **Total (n)** |
| No pretreatment* | 5 | 28 | 1 | **34** | 7 | 10 | 1 | **18** |
| HU* | 3 | 6 | 0 | **9** | 3 | 8 | 0 | **11** |
| IFNa | 0 | 0 | 0 | **0** | 2 | 2 | 0 | **4** |
| HU and IFNa | 0 | 0 | 0 | **0** | 1 | 0 | 0 | **1** |
| **Total** | **8** | **34** | **1** | **43** | **13** | **20** | **1** | **34** |
|  |  |  |  |  |  |  |  |  |
| *Alternative response criteria #5 (blood counts and splenomegaly)* | NR (n) | PR (n) | CR (n) | **Total (n)** | NR (n) | PR (n) | CR (n) | **Total (n)** |
| No pretreatment^$^ | 5 | 22 | 7 | **34** | 6 | 8 | 4 | **18** |
| HU^$^ | 3 | 4 | 2 | **9** | 3 | 4 | 4 | **11** |
| IFNa | 0 | 0 | 0 | **0** | 2 | 2 | 0 | **4** |
| HU and IFNa | 0 | 0 | 0 | **0** | 1 | 0 | 0 | **1** |
| **Total** | **8** | **26** | **9** | **43** | **12** | **14** | **8** | **34** |
|  |  |  |  |  |  |  |  |  |

RUX: Ruxolitinib. BAT: Best available therapy. HU: Hydroxyurea. IFNa: Interferon-alpha. NR: No response. *No difference in responses (criteria #1) between no pretreatment and HU pretreatment was detected by Fisher´s Exact Testing (p=0.475 for RUX, p=0.807 for BAT). ^$^No difference in responses (criteria #5) between no pretreatment and HU pretreatment was detected by Fisher´s Exact Testing (p=0.378 for RUX, p=0.807 for BAT).

**Supplementary Figure 4 (Fig. S4):** Responses at 6 months separated for the type of BAT treatment.

| **Type of BAT treatment** | **Response** | | | |
| --- | --- | --- | --- | --- |
| *Strict response criteria #1 (symptom threshold = 0)* | NR (n) | PR (n) | CR (n) | **Total (n)** |
| Hydroxyurea* | 7 | 14 | 1 | **22** |
| IFNa* | 1 | 2 | 0 | **3** |
| Other therapy | 1 | 1 | 0 | **2** |
| No drug therapy (watchful waiting) | 0 | 2 | 0 | **2** |
| **Total** | **9** | **19** | **1** | **29** |
|  |  |  |  |  |
| *Alternative response criteria #5 (blood counts and splenomegaly)* | NR (n) | PR (n) | CR (n) | **Total (n)** |
| Hydroxyurea^$^ | 6 | 10 | 6 | **22** |
| IFNa^$^ | 1 | 1 | 1 | **3** |
| Other therapy | 1 | 0 | 1 | **2** |
| No drug therapy (watchful waiting) | 0 | 2 | 0 | **2** |
| **Total** | **8** | **13** | **8** | **29** |
|  |  |  |  |  |

RUX: Ruxolitinib. BAT: Best available therapy. IFNa: Interferon-alpha. HU: Hydroxyurea. NR: No response. *No difference in responses (criteria #1) between HU and IFNa was detected by Fisher´s Exact Testing (p>0.999). ^$^No difference in responses (criteria #5) between HU and IFNa was detected by Fisher´s Exact Testing (p>0.999).

**Supplementary Fig. 5 (Table S5):** Number of patients with adverse events (AE) during the first 24 months

| **MEDDRA > System organ class**  **& CTC-Grade <3 vs. >=3** | Total Count | % of (n=77) | # Events | Rux Count | % of (n=43) | # Events | BAT Count | % of (n=17) | # Events | BAT-CO Count | % of (n=17) | # Events | p-value |
| --- | --- | --- | --- | --- | --- | --- | --- | --- | --- | --- | --- | --- | --- |
| ANY (S)AE | 65 | 84,4 | 577 | 40 | 93.0 | 329 | 10 | 58.8 | 86 | 15 | 88.2 | 162 | **0.006** |
| & CTC-Grade <3 | 26 | 33,8 | 507 | 13 | 30.2 | 285 | 8 | 47.1 | 81 | 5 | 29.4 | 141 | . |
| & CTC-Grade >=3 | 39 | 50,6 | 70 | 27 | 62.8 | 44 | 2 | 11.8 | 5 | 10 | 58.8 | 21 | . |
| MEDDRA | 65 | 84,4 | 577 | 40 | 93.0 | 329 | 10 | 58.8 | 86 | 15 | 88.2 | 162 | **0.006** |
| General disorders and administration site conditions | 32 | 41,6 | 46 | 16 | 37.2 | 25 | 6 | 35.3 | 8 | 10 | 58.8 | 13 | 0.310 |
| & CTC-Grade <3 | 31 | 40,3 | 45 | 16 | 37.2 | 25 | 6 | 35.3 | 8 | 9 | 52.9 | 12 | . |
| & CTC-Grade >=3 | 1 | 1,3 | 1 | . | 0.0 | . | . | 0.0 | . | 1 | 5.9 | 1 | . |
| Eye disorders | 6 | 7,8 | 10 | 5 | 11.6 | 9 | . | 0.0 | . | 1 | 5.9 | 1 | 0.397 |
| & CTC-Grade <3 | 5 | 6,5 | 8 | 4 | 9.3 | 7 | . | 0.0 | . | 1 | 5.9 | 1 | . |
| & CTC-Grade >=3 | 1 | 1,3 | 2 | 1 | 2.3 | 2 | . | 0.0 | . | . | 0.0 | . | . |
| Surgical and medical procedures | 4 | 5,2 | 4 | 4 | 9.3 | 4 | . | 0.0 | . | . | 0.0 | . | 0.497 |
| & CTC-Grade <3 | 2 | 2,6 | 2 | 2 | 4.7 | 2 | . | 0.0 | . | . | 0.0 | . | . |
| & CTC-Grade >=3 | 2 | 2,6 | 2 | 2 | 4.7 | 2 | . | 0.0 | . | . | 0.0 | . | . |
| Respiratory, thoracic and mediastinal disorders | 16 | 20,8 | 20 | 6 | 14.0 | 7 | 3 | 17.6 | 3 | 7 | 41.2 | 10 | 0.074 |
| & CTC-Grade <3 | 14 | 18,2 | 18 | 5 | 11.6 | 6 | 3 | 17.6 | 3 | 6 | 35.3 | 9 | . |
| & CTC-Grade >=3 | 2 | 2,6 | 2 | 1 | 2.3 | 1 | . | 0.0 | . | 1 | 5.9 | 1 | . |
| Reproductive system and breast disorders | 2 | 2,6 | 2 | 1 | 2.3 | 1 | . | 0.0 | . | 1 | 5.9 | 1 | 0.691 |
| & CTC-Grade <3 | 2 | 2,6 | 2 | 1 | 2.3 | 1 | . | 0.0 | . | 1 | 5.9 | 1 | . |
| Skin and subcutaneous tissue disorders | 28 | 36,4 | 44 | 15 | 34.9 | 25 | 6 | 35.3 | 11 | 7 | 41.2 | 8 | 0.948 |
| & CTC-Grade <3 | 27 | 35,1 | 43 | 14 | 32.6 | 24 | 6 | 35.3 | 11 | 7 | 41.2 | 8 | . |
| & CTC-Grade >=3 | 1 | 1,3 | 1 | 1 | 2.3 | 1 | . | 0.0 | . | . | 0.0 | . | . |
| Renal and urinary disorders | 5 | 6,5 | 7 | 3 | 7.0 | 4 | 1 | 5.9 | 2 | 1 | 5.9 | 1 | 1.000 |
| & CTC-Grade <3 | 3 | 3,9 | 4 | 2 | 4.7 | 3 | . | 0.0 | . | 1 | 5.9 | 1 | . |
| & CTC-Grade >=3 | 2 | 2,6 | 3 | 1 | 2.3 | 1 | 1 | 5.9 | 2 | . | 0.0 | . | . |
| Blood and lymphatic system disorders | 10 | 13,0 | 21 | 4 | 9.3 | 7 | . | 0.0 | . | 6 | 35.3 | 14 | **0.009** |
| & CTC-Grade <3 | 9 | 11,7 | 20 | 4 | 9.3 | 7 | . | 0.0 | . | 5 | 29.4 | 13 | . |
| & CTC-Grade >=3 | 1 | 1,3 | 1 | . | 0.0 | . | . | 0.0 | . | 1 | 5.9 | 1 | . |
| Gastrointestinal disorders | 34 | 44,2 | 68 | 17 | 39.5 | 30 | 7 | 41.2 | 11 | 10 | 58.8 | 27 | 0.474 |
| & CTC-Grade <3 | 25 | 32,5 | 57 | 14 | 32.6 | 27 | 6 | 35.3 | 10 | 5 | 29.4 | 20 | . |
| & CTC-Grade >=3 | 9 | 11,7 | 11 | 3 | 7.0 | 3 | 1 | 5.9 | 1 | 5 | 29.4 | 7 | . |
| Immune system disorders | 1 | 1,3 | 1 | 1 | 2.3 | 1 | . | 0.0 | . | . | 0.0 | . | 1.000 |
| & CTC-Grad >=3 | 1 | 1,3 | 1 | 1 | 2.3 | 1 | . | 0.0 | . | . | 0.0 | . | . |
| Nervous system disorders | 37 | 48,1 | 69 | 17 | 39.5 | 32 | 9 | 52.9 | 11 | 11 | 64.7 | 26 | 0.208 |
| & CTC-Grade <3 | 32 | 41,6 | 64 | 15 | 34.9 | 30 | 9 | 52.9 | 11 | 8 | 47.1 | 23 | . |
| & CTC-Grade >=3 | 5 | 6,5 | 5 | 2 | 4.7 | 2 | . | 0.0 | . | 3 | 17.6 | 3 | . |
| Ear and labyrinth disorders | 5 | 6,5 | 8 | 4 | 9.3 | 6 | . | 0.0 | . | 1 | 5.9 | 2 | 0.819 |
| & CTC-Grad <3 | 5 | 6,5 | 8 | 4 | 9.3 | 6 | . | 0.0 | . | 1 | 5.9 | 2 | . |
| Vascular disorders | 16 | 20,8 | 17 | 9 | 20.9 | 10 | 2 | 11.8 | 2 | 5 | 29.4 | 5 | 0.484 |
| & CTC-Grade <3 | 11 | 14,3 | 12 | 6 | 14.0 | 7 | 1 | 5.9 | 1 | 4 | 23.5 | 4 | . |
| & CTC-Grade >=3 | 5 | 6,5 | 5 | 3 | 7.0 | 3 | 1 | 5.9 | 1 | 1 | 5.9 | 1 | . |
| Neoplasms benign, malignant and unspecified | 8 | 10,4 | 9 | 5 | 11.6 | 6 | . | 0.0 | . | 3 | 17.6 | 3 | 0.273 |
| & CTC-Grad <3 | 4 | 5,2 | 4 | 2 | 4.7 | 2 | . | 0.0 | . | 2 | 11.8 | 2 | . |
| & CTC-Grad >=3 | 4 | 5,2 | 5 | 3 | 7.0 | 4 | . | 0.0 | . | 1 | 5.9 | 1 | . |
| Cardiac disorders | 6 | 7,8 | 8 | 3 | 7.0 | 5 | 1 | 5.9 | 1 | 2 | 11.8 | 2 | 0.850 |
| & CTC-Grade <3 | 3 | 3,9 | 5 | 1 | 2.3 | 3 | . | 0.0 | . | 2 | 11.8 | 2 | . |
| & CTC-Grade >=3 | 3 | 3,9 | 3 | 2 | 4.7 | 2 | 1 | 5.9 | 1 | . | 0.0 | . | . |
| Infections and infestations | 32 | 41,6 | 57 | 17 | 39.5 | 28 | 6 | 35.3 | 11 | 9 | 52.9 | 18 | 0.635 |
| & CTC-Grade <3 | 27 | 35,1 | 51 | 14 | 32.6 | 24 | 6 | 35.3 | 11 | 7 | 41.2 | 16 | . |
| & CTC-Grade >=3 | 5 | 6,5 | 6 | 3 | 7.0 | 4 | . | 0.0 | . | 2 | 11.8 | 2 | . |
| Psychiatric disorders | 10 | 13,0 | 14 | 5 | 11.6 | 6 | 2 | 11.8 | 2 | 3 | 17.6 | 6 | 0.897 |
| & CTC-Grade <3 | 8 | 10,4 | 12 | 4 | 9.3 | 5 | 2 | 11.8 | 2 | 2 | 11.8 | 5 | . |
| & CTC-Grade >=3 | 2 | 2,6 | 2 | 1 | 2.3 | 1 | . | 0.0 | . | 1 | 5.9 | 1 | . |
| Musculoskeletal and connective tissue disorders | 25 | 32,5 | 47 | 15 | 34.9 | 25 | 3 | 17.6 | 6 | 7 | 41.2 | 16 | 0.298 |
| & CTC-Grade <3 | 22 | 28,6 | 44 | 15 | 34.9 | 25 | 3 | 17.6 | 6 | 4 | 23.5 | 13 | . |
| & CTC-Grade >=3 | 3 | 3,9 | 3 | . | 0.0 | . | . | 0.0 | . | 3 | 17.6 | 3 | . |
| Metabolism and nutrition disorders | 13 | 16,9 | 19 | 10 | 23.3 | 14 | 1 | 5.9 | 3 | 2 | 11.8 | 2 | 0.275 |
| & CTC-Grad <3 | 11 | 14,3 | 16 | 8 | 18.6 | 11 | 1 | 5.9 | 3 | 2 | 11.8 | 2 | . |
| & CTC-Grad >=3 | 2 | 2,6 | 3 | 2 | 4.7 | 3 | . | 0.0 | . | . | 0.0 | . | . |
| Investigations | 19 | 24,7 | 95 | 15 | 34.9 | 78 | 2 | 11.8 | 13 | 2 | 11.8 | 4 | 0.078 |
| & CTC-Grad <3 | 12 | 15,6 | 83 | 8 | 18.6 | 66 | 2 | 11.8 | 13 | 2 | 11.8 | 4 | . |
| & CTC-Grad >=3 | 7 | 9,1 | 12 | 7 | 16.3 | 12 | . | 0.0 | . | . | 0.0 | . | . |
| Injury. poisoning and procedural complications | 10 | 13,0 | 11 | 5 | 11.6 | 6 | 2 | 11.8 | 2 | 3 | 17.6 | 3 | 0.897 |
| & CTC-Grade <3 | 8 | 10,4 | 9 | 3 | 7.0 | 4 | 2 | 11.8 | 2 | 3 | 17.6 | 3 | . |
| & CTC-Grade >=3 | 2 | 2,6 | 2 | 2 | 4.7 | 2 | . | 0.0 | . | . | 0.0 | . | . |

^a^Fisher´s exact test RUX vs. BAT vs. BAT-CO (without correction for multiple testing, BAT-CO includes AEs under BAT and under RUX). CTC: Common toxicity criteria. AE: Adverse event. SAE: Serious AE. In detail, the blood and lymphatic disorders comprised anemia (n=6), leukocytopenia (n=5), thrombocytopenia (n=5), splenic infarction (n=1), coagulopathy (n=1), leukocytosis (n=1), lymphadenopathy (n=1), and neutropenia (n=1). The respiratory, thoracic and mediastinal disorders included cough (n=5), epistaxis (n=3), exertional dyspnea (n=3), dyspnea (n=2), oropharyngeal pain (n=2), asthma (n=1), hemothorax (n=1), peritonsillar abscess (n=1), pleural effusion (n=1), and the three most frequently occurring investigations were increased gamma-glutamyltransferase (n=15), increased lipase (n=13), and increased blood creatine phosphokinase (n=12).

**Supplementary Figure 6 (Fig. S6):** Blood counts and spleen response at month 24.

**Supplementary Figure 7 (Fig. S7): A-C:** Intra-patient development of pruritus from baseline to end of month 24 (EOM24). **D:** Variant allele frequency of JAK2V617F at baseline (BL) or, if no BL value was available, end of month 1 (M1), and at month 24 (M24). ****p<0.001, *p=0.0498. RUX (n=37): Ruxolitinib, BAT (n=13): Best available therapy, BAT-CO (n=14): Crossover from BAT to RUX.

**D**

**Supplementary Figure 8 (Fig. S8):** Responses at 6 months separated for mean daily dose of ruxolitinib treatment.

| **Mean daily dose of ruxolitinib** | **Response** | | | |
| --- | --- | --- | --- | --- |
| *Strict response criteria #1 (symptom threshold = 0)* | NR  n (%) | PR  n (%) | CR  n (%) | **Total (n)** |
| Ruxolitinib dose < 20mg/d | 1 (17) | 5 (83) | 0 (0) | **6** |
| Ruxolitinib dose = 20mg/d | 3 (15) | 16 (80) | 1 (5) | **20** |
| Ruxolitinib dose > 20mg/d | 4 (24) | 13 (77) | 0 (0) | **17** |
| **Total** | **8** | **34** | **1** | **43** |
|  |  |  |  |  |
| *Alternative response criteria #5 (blood counts and splenomegaly)* | NR  n (%) | PR  n (%) | CR  n (%) | **Total (n)** |
| Ruxolitinib dose < 20mg/d | 1 (17) | 3 (50) | 2 (33) | **6** |
| Ruxolitinib dose = 20mg/d | 3 (15) | 11 (55) | 6 (30) | **20** |
| Ruxolitinib dose > 20mg/d | 4 (24) | 12 (71) | 1 (6) | **17** |
| Total | 8 | 26 | 9 | **43** |
|  |  |  |  |  |

d: daily; NR: No response.

**Supplementary Figure 9 (Fig. S9):** Time (days) from PV diagnosis to baseline visit (first study treatment).
